# Palmitic acid conjugation enhances potency of tricyclo-DNA splice switching oligonucleotides

**DOI:** 10.1093/nar/gkab1199

**Published:** 2021-12-10

**Authors:** Karima Relizani, Lucía Echevarría, Faouzi Zarrouki, Cécile Gastaldi, Chloe Dambrune, Philippine Aupy, Adrian Haeberli, Marek Komisarski, Thomas Tensorer, Thibaut Larcher, Fedor Svinartchouk, Cyrille Vaillend, Luis Garcia, Aurélie Goyenvalle

**Affiliations:** Université Paris-Saclay, UVSQ, Inserm, END-ICAP, 78000 Versailles, France; SQY Therapeutics, UVSQ, 78180 Montigny le Bretonneux, France; Université Paris-Saclay, UVSQ, Inserm, END-ICAP, 78000 Versailles, France; SQY Therapeutics, UVSQ, 78180 Montigny le Bretonneux, France; Université Paris-Saclay, UVSQ, Inserm, END-ICAP, 78000 Versailles, France; Université Paris-Saclay, CNRS, Institut des Neurosciences Paris Saclay, 91190, Gif-sur-Yvette, France; LIA BAHN, centre scientifique de Monaco, 98000, Monaco; Université Paris-Saclay, UVSQ, Inserm, END-ICAP, 78000 Versailles, France; Université Paris-Saclay, UVSQ, Inserm, END-ICAP, 78000 Versailles, France; SYNTHENA AG, Bern, Switzerland; SYNTHENA AG, Bern, Switzerland; SQY Therapeutics, UVSQ, 78180 Montigny le Bretonneux, France; SYNTHENA AG, Bern, Switzerland; INRAE Oniris, UMR 703 PAnTher, Nantes, France; SQY Therapeutics, UVSQ, 78180 Montigny le Bretonneux, France; Université Paris-Saclay, CNRS, Institut des Neurosciences Paris Saclay, 91190, Gif-sur-Yvette, France; Université Paris-Saclay, UVSQ, Inserm, END-ICAP, 78000 Versailles, France; LIA BAHN, centre scientifique de Monaco, 98000, Monaco; Université Paris-Saclay, UVSQ, Inserm, END-ICAP, 78000 Versailles, France; LIA BAHN, centre scientifique de Monaco, 98000, Monaco

## Abstract

Tricyclo-DNA (tcDNA) is a conformationally constrained oligonucleotide analog that has demonstrated great therapeutic potential as antisense oligonucleotide (ASO) for several diseases. Like most ASOs in clinical development, tcDNA were modified with phosphorothioate (PS) backbone for therapeutic purposes in order to improve their biodistribution by enhancing association with plasma and cell protein. Despite the advantageous protein binding properties, systemic delivery of PS-ASO remains limited and PS modifications can result in dose limiting toxicities in the clinic. Improving extra-hepatic delivery of ASO is highly desirable for the treatment of a variety of diseases including neuromuscular disorders such as Duchenne muscular dystrophy. We hypothesized that conjugation of palmitic acid to tcDNA could facilitate the delivery of the ASO from the bloodstream to the interstitium of the muscle tissues. We demonstrate here that palmitic acid conjugation enhances the potency of tcDNA-ASO in skeletal and cardiac muscles, leading to functional improvement in dystrophic mice with significantly reduced dose of administered ASO. Interestingly, palmitic acid-conjugated tcDNA with a full phosphodiester backbone proved effective with a particularly encouraging safety profile, offering new perspectives for the clinical development of PS-free tcDNA-ASO for neuromuscular diseases.

## INTRODUCTION

Antisense Oligonucleotides (ASOs)-based therapeutics have made tremendous progress in the past few decades and represent very attractive drugs for a variety of diseases. Several ASOs have been approved for use in the clinic and many more are in the preclinical or clinical pipeline. However, systemically delivered ASOs mostly distribute to liver, kidney and spleen whilst other tissues of interest such as skeletal and cardiac muscles remain challenging targets. This is exemplified by the discontinuation of several clinical programs for myotonic dystrophy type I (DM1) (1) and Duchenne muscular dystrophy (DMD) using various chemistries (2). Most ASOs in clinical development are modified using the phosphorothioate (PS) backbone which improves metabolic stability and allows association with plasma and cell surface proteins (3,4). PS-ASOs have been shown to reach muscle tissues in mouse models of muscle disease but the doses required are generally much higher than those needed for other organs like liver, which may result in dose-limiting toxicities in the clinic as seen for drisapersen (a 2’-*O*-methyl-PS ASO developed for DMD therapy). While several neuromuscular diseases (NMD) are particularly amenable for ASO-based treatment, clinical development for these disorders has proven difficult mostly due to the abundance of skeletal muscle and the poor distribution to muscle tissues. Apart from the evident success of nusinersen, a 2’-*O*-methoxyethyl (2’MOE) ASO which is administered intrathecally to treat spinal muscular atrophy (SMA), the development of nucleic acid therapy for NMDs has been accompanied by several failures, highlighting problems of safety, efficacy, and tissue targeting that need to be overcome (2). Moreover, the mechanism of action of ASO for NMDs which is often splice switching, requires localization of ASO in the nucleus in contrast with RNA degradation mechanisms, adding another delivery challenge.

Extra-hepatic delivery of ASOs is an active line of research, where various backbone modifications and different types of conjugates are being evaluated (5). For example conjugation of ASOs to cell-penetrating peptides or antibodies targeting specific receptors (such as the transferrin receptor) has shown promising results (2). Phosphorodiamidate morpholino (PMO) are so far the only ASO approved for the systemic treatment of DMD but the functional benefit in DMD patients is still minimal. Preliminary results from a phase 2 clinical trial in DMD patients using peptide conjugated PMO (PPMO) revealed higher levels of dystrophin restoration than with their naked PMO counterpart (https://investorrelations.sarepta.com/news-releases/news-release-details/sarepta-therapeutics-reports-positive-clinical-results-phase-2).

Among developments of alternative chemistries, we have previously demonstrated that tricyclo-DNA (tcDNA)-ASOs display unprecedented uptake in many tissues including cardiac muscle and the central nervous system (CNS) after systemic administration in mouse models of DMD (6,7) and SMA (8). The antisense strategy for DMD aims to eliminate one or several exons, by masking key splicing sites, to restore the reading frame and generate a shorter but functional dystrophin. Systemic delivery of full PS-tcDNA allows restoration of dystrophin in skeletal and cardiac muscles and to a lower extent in the brain of dystrophic mice, leading to muscle function improvement and correction of behavioral features linked to the loss of brain dystrophin. Still, as for all PS-ASOs, high doses of PS-tcDNA may lead to undesirable effects mainly due to their capacity to bind plasma proteins (9). Effects of PS backbones may include immune cell activation (10), complement activation (mostly reported in monkey studies) (11) or prolongation of clotting times (9,12), known to normalize as ASOs are cleared from the bloodstream. In particular it has been shown that low-level, but sustained, complement activation may lead to depletion of complement and damage to the vascular system and kidney (13,14). Moreover, the increased association between PS-ASO and proteins prevents their rapid excretion in the urine as they form larger complexes that impair their filtration by the glomerulus. Therefore, repeated systemic administrations of PS-ASOs are likely to cause cellular toxicity in typical target organs like kidney and liver, where they accumulate. In this context, development of PS-free ASOs is an attractive alternative particularly for tcDNAs which are stable in their full phosphodiester (PO) version as opposed to other phosphodiester based ASOs which require PS bonds for stability (15,16).

Unfortunately, when exploring the potential of minimizing PS content, we previously demonstrated that tcDNA-PO display poor biodistribution and efficacy (17). In order to compensate the lack of PS linkages which are known to bind serum albumin with dissociation constants in the low micromolar range (18), we investigated the conjugation of tcDNA-ASO to palmitic acid. We hypothesized that palmitic acid could facilitate tcDNA-ASO transport across the continuous capillary endothelium in the skeletal and cardiac muscle through improved binding of the conjugated-tcDNA to serum albumin (19).

In this study, we describe the impact of palmitic acid conjugation to tcDNA-ASOs, in their full phosphodiester (PO) or full phosphorothioate (PS) versions, using the DMD mouse (*mdx*) as model. We demonstrate that these conjugates show enhanced potency in skeletal and cardiac muscles, which allows a significant reduction of the administered dose. We also report the functional improvement in dystrophic mice and the safety profile following a long-term treatment with these conjugated compounds as well as the persistence of the treatment efficacy after a recovery period. Overall, our data suggest that palmitic acid conjugation can safely and effectively substitute for PS linkages offering new perspectives for the clinical development of PS-free tcDNA-ASO.

## MATERIALS AND METHODS

### Antisense oligonucleotides and animal experiments

Animal procedures were performed in accordance with national and European legislation, approved by the French government (Ministère de l’Enseignement Supérieur et de la Recherche, Autorisation APAFiS #6518). *Mdx* (C57BL/10ScSn-Dmd^mdx^/J) mice were bred in our animal facility at the Plateforme 2Care, UFR des Sciences de la santé, Université de Versailles Saint Quentin and were maintained in a standard 12-h light/dark cycle with free access to food and water. Mice were weaned at weeks 4–5 postnatal and 2–5 individuals were housed per cage.

**Figure 1.**
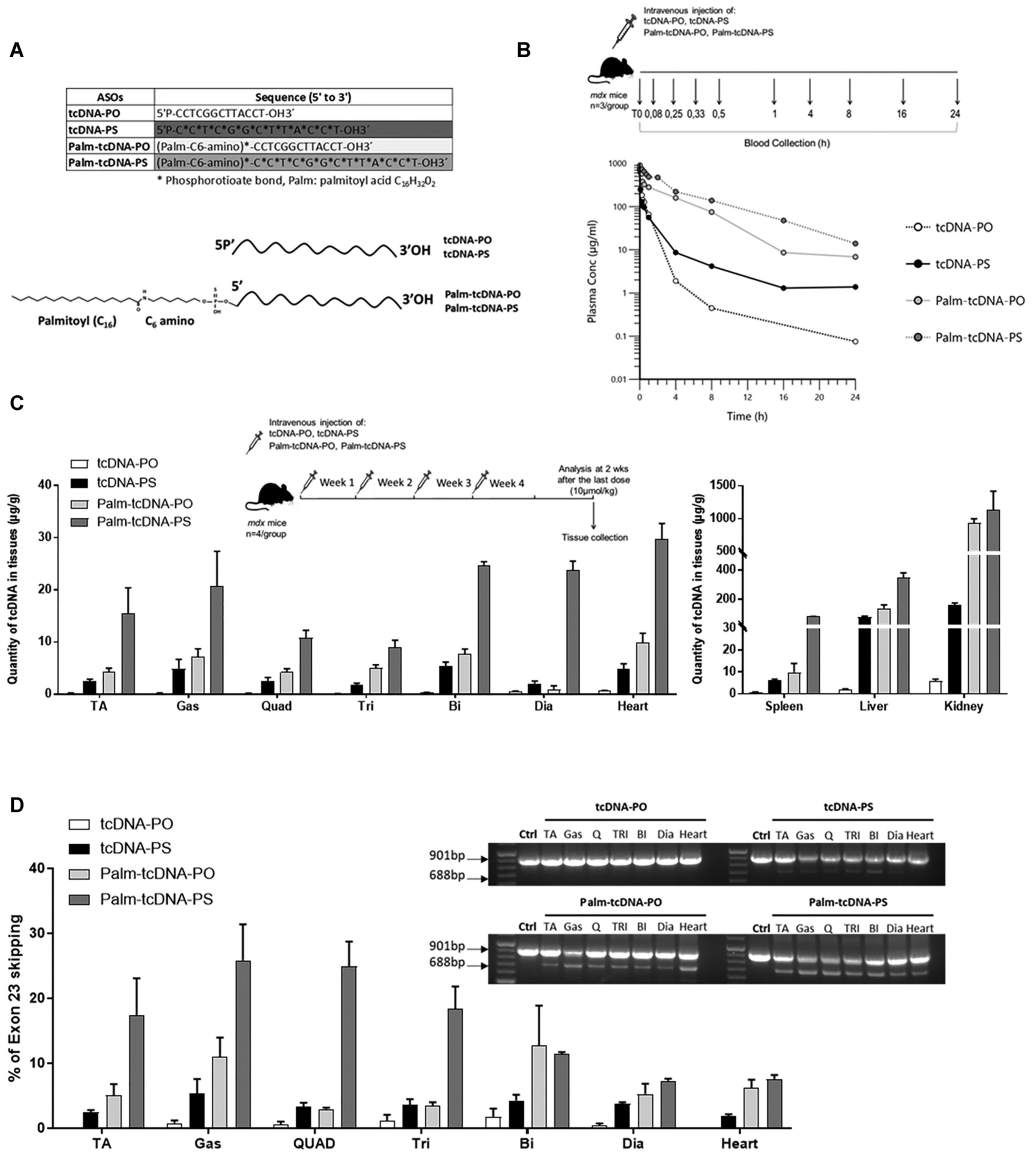
Palmitic acid conjugation enhances the biodistribution and efficacy of tcDNA-ASO. (**A**) Sequences of the different tcDNA-ASOs and schematic design representing the conjugation of the palmitic acid at the 5’ end of tcDNA (tcDNA-PO, tcDNA-PS) using a C6-amino linker and a PS bond. (**B**) Experimental pharmacokinetics protocol (upper panel): *mdx* mice received a single intravenous dose of 10 μmol/kg of unconjugated (tcDNA-PO or tcDNA-PS) or conjugated tcDNA (palm-tcDNA-PO or palm-tcDNA-PS) and blood samples were collected at different time points (*t* = 0, 0.08 h (i.e. 5 min), 0.33 h (i.e. 15 min), 0.5 h (i.e. 30 min), 1, 4, 8, 16 and 24 h) after the administration. Semi-log plots of serum concentration of each tcDNA-ASO versus time from mice treated with 10 μmol/kg of unconjugated (tcDNA-PO or tcDNA-PS) or conjugated tcDNA (palm-tcDNA-PO or palm-tcDNA-PS). Data are represented as mean, *n* = 3/time point). (**C**) Quantification of tcDNA-ASO content in various tissues by fluorescent hybridization assay following a 4-week treatment at the dose of 10 μmol/kg/week. D) Detection of exon 23–skipped dystrophin mRNA by nested RT-PCR (right) and quantification of exon 23 skipping levels by qRT-PCR (bottom) in the different muscle tissues following a 4-week treatment at the dose of 10 μmol/kg/week (TA: tibialis anterior, gas: gastrocnemius, quad: quadriceps, Tri: triceps, Bi: biceps; Dia: diaphragm). Results are expressed as mean ± SEM; *n* = 4 mice per group.

All tcDNA-ASO targeting the donor splice site of exon 23 of the mouse dystrophin pre-mRNA, used in this study are detailed in Figure 1A and were synthesized by SYNTHENA (Bern, Switzerland). Palmitic acid was conjugated at the 5’end of tcDNA-PO or tcDNA-PS ASO via a C6-amino linker and a phosphorothioate bond.

For pharmacokinetics studies, 6–8 week-old *mdx* mice received a single intravenous injection of 10 μmol/kg of unconjugated (tcDNA-PO or tcDNA-PS) or conjugated tcDNA (palm-tcDNA-PO or palm-tcDNA-PS) under general anesthesia using 1.5–2% isoflurane. Blood samples were collected at different time points (*t* = 0, 5 min, 15 min, 30 min, 1 h, 4 h, 8 h, 16 h and 24 h) after the administration (*n* = 3/time point). Pharmacokinetics was analysed using WinNonlin 8.1 software (Pharsight Corporation, Mountain View, CA). Semilogarithmic plots of tcDNA-ASOs serum level means versus time indicated biexponential decrease.

For repeated injection protocol, 6–8 week-old *mdx* mice were injected intravenously, under general anesthesia using 1.5–2% isoflurane, once a week with the corresponding tcDNA-ASO for a period of 4–12 weeks. Age-matched *mdx* groups receiving an equivalent volume of sterile saline were included as controls and C57BL/10 mice were included as wild-type controls. One hour after the first injection, blood samples were collected to measure complement C3. Additional blood samples were collected at the end of the treatment when animals were euthanized for MYOM-3 and biochemistry analysis. Animals were euthanized 2 weeks or 12 weeks after the last injection and muscles and tissues were harvested and snap-frozen in liquid nitrogen–cooled isopentane and stored at −80°C before further analysis. To assess the safety of the palm-tcDNA-ASO treatment, liver and kidney were sampled at the end of the protocol (2 weeks or 12 weeks after the last dose), fixed in 10% neutral buffered formalin, and embedded in paraffin wax. 5-μm-thick sections were then stained with routine hematoxylin-eosin-saffron (HES) for histopathological evaluation which was further performed by a veterinary pathologist blind to treatment. Hepatocytic nuclear pleomorphism and atypias were scored on a scale of 0 to 3 (0 = no lesion, 1 = mild pleomorphism, 2 = mild pleomorphism with some atypias, 3 = severe pleomorphism with trabecular disorganization).

Sample sizes and n values are indicated in each figure legend. Investigators were blinded for RNA and protein analysis.

### TcDNA quantification by fluorescent hybridization assay and LCMS

Tissues were homogenized using the Precellys 24 (Bertin Instruments, France) in lysis buffer (100 mmol/l Tris–HCl, pH 8.5, 200 mmol/l NaCl, 5 mmol/l EDTA, 0.2% sodium dodecyl sulfate) containing 2 mg/ml of proteinase K (Invitrogen) (50 mg tissue/ ml of buffer), followed by incubation overnight at 55°C in a hybridization oven. After centrifugation at 14 000 rpm (Sorval ST 8R centrifuge, 75005719 rotor) for 15 min, the supernatant was used in the assay. Quantification of tcDNA was performed using a hybridization assay with a molecular beacon probe, as previously described (17). Briefly, 10 μl of tissue lysates or serum were incubated with a 5’ Cy3-DNA complementary probe conjugated with HBQ quencher at 3’ in a black non-binding 96-well plates (Fischer Scientific). PBS was added to a final volume of 100 μl per well and fluorescence was measured on a spectrophotometer (Ex 544 nm/Em 590 nm using FluoStar Omega). The amount of tcDNA in tissues was determined using a standard curve build on the measurement of known tcDNA quantities dissolved in the respective tissue lysates of mock-injected animals.

Quantification of tcDNA-ASO was also performed by LC/MS analysis on an Agilent 1100 HPLC system coupled to an Agilent 6530 TOF-MS mass spectrometer. Standard curves of each ASO were established in Milli-Q water (2μM) with 3 different injection volumes (2, 4, 10 μl). TcDNA-ASO from tissue lysate (50 μl) was extracted by solid-phase extraction (SPE) using Oasis HLB 1cc (10 mg) Extraction Cartridges (Waters). Eluate was collected in a single 1.5 ml Eppendorf tube and lyophilized to dryness in a speed-vac. 50 μl of Milli-Q water was added to the lyophilized sample. Mixture was transferred to a VWR HPLC vial with inlet and injected on LC/MS. Briefly, separation was accomplished using an HPLCMS system consisting of a quaternary pump, UV detector, a column oven, an autosampler and a Q-TOF mass spectrometer. An ion-pair method was applied. Samples were injected on an ACQUITY UPLC Oligonucleotide BEH C18 Column (130Å, 1.7 μm, 2.1 mm × 100 mm, Waters). The column was maintained at 75°C. A buffer (400 mM HFIP, 15 mM TEA, + 10% Methanol) prepared in advance and Methanol were used as the mobile phase at a flow rate of 0.2 ml/min. Two gradients were performed in the same analysis method. The first one was used to separate potential metabolites that have lost 5’end palmitoyl moiety. Methanol was increased from 5% to 25% over 19min. The second gradient was used to isolate the palmitoyl conjugated-ASO. Methanol was increased from 50% to 60% over 9min.

Manual evaluation was performed by comparing a table of calculated *m*/*z* values corresponding to potential metabolites with the peaks present in TIC chromatograms. Peak areas from UV chromatograms were determined for ASOs and concentrations were calculated using the calibration curves. As a control and for really low values, quantifications of ASOs in tissues were also determined from extracted ion chromatograms (EIC).

Plasma pharmacokinetics were compared by non-compartmental analysis via sparse sampling design using Phoenix Winnonlin 8.1 (Pharsight, Mountain View, CA).

### Serum and urine analysis

Analyses of serum creatine kinase (CK), alanine aminotransferase (ALT), aspartate aminotransferase (AST), alkaline phosphatase (ALP), bilirubin, creatinine, urea and albumin levels were performed by the pathology laboratory at Mary Lyon Centre, Medical Research Council, Harwell, Oxfordshire, UK.

Coagulation assays: human plasma from healthy volunteers was obtained from the French Blood Donors Organization (EFS, Etablissement Français du Sang). Mouse or Human citrated plasma samples were incubated *in vitro* with 2 mg/ml of tcDNA for 20 min at 37°C, then prothrombin time (PT) and activated partial thromboplastin time (aPTT) assays were performed on a semi-automated START max coagulometer (Stago) following manufacturer's instructions.

For *in vitro* complement activation studies, tcDNA was incubated with mouse or human serum at 37°C for 45 min. Determination of complement activation was evaluated using mouse C3a elisa kit (Teco Medical, Switzerland) and human C3aPlus kit (Quidel Co., San Diego CA, USA). 5 mg/ml Zymosan (Complement Technology, Inc, Texas, USA) was used as positive control.

Urines were collected using metabolic cages over 24 h, directly in refrigerated tubes (4°C). Upon collection, urines were centrifuged at 10 000 g for 10 min and supernatants were aliquots were frozen at –80°C for further analysis. Urine creatinine was measured using Creatinine assay kit (R&D Systems, Inc, Minneapolis, MN) following manufacturer's instructions. Total protein in urine samples was measured as previously described (20). Briefly, proteins were precipitated from urine samples by adding 40 μl dH_2_O and 200 μl of prechilled acetone to 10 μl of urine. Samples were then incubated at –20°C for 30 min, then centrifuged at 14 000 g, 4°C for 15 min. Pellets were resuspended in 40 μl dH_2_O and protein concentration was measured using Pierce BCA assay (Thermo Scientific, Rockford, IL). Albumin from urine samples was measured using the albumin ELISA kit (Bethy Laboratories, Montgomery, TX) following manufacturer's instructions. Acute kidney injury (AKI) biomarkers levels were analysed by multiplex assays, using the Luminex® technology. The multiplex kidney injury panels (MKI1MAG-94K, MKI2MAG-94K, Merck-Millipore) were used according to the manufacturer's instructions to measure levels of β-2-microglobulin (B2M), Renin, Kidney Injury Molecule 1 (KIM-1), interferon-gamma induced protein 10 (IP-10), Vascular endothelial growth factor (VEGF), Cystatin C, epidermal growth factor (EGF), Lipocalin-2-NGAL, Clusterin and Osteopontin (OPN). The results were read using a Bio-Plex MAGPIX Multiplex reader and analysed with the Bio-Plex manager 6.1 software (Bio-Rad, France).

### RNA analysis

Total RNA was isolated from intervening muscle sections collected during cryosection using TRIzol reagent according to the manufacturer's instructions (Thermo Fisher Scientific, USA). Aliquots of 500 ng of total RNA were used for RT-PCR analysis using the Access RT-PCR System (Promega, USA) in a 50 μl reaction using the external primers Ex 20Fo (5′-CAGAATTCTGCCAATTGCTGAG-3′) and Ex 26Ro (5′-TTCTTCAGCTTGTGTCATCC-3′). The cDNA synthesis was carried out at 45°C for 45 min, directly followed by the primary PCR of 30 cycles of 95°C (30 s), 55°C (1 min) and 72°C (2 min). 2 μl of these reactions were then re-amplified in nested PCRs by 22 cycles of 95°C (30 s), 55°C (1 min) and 72°C (2 min) using the internal primers Ex 20Fi (5′-CCCAGTCTACCACCCTATCAGAGC-3′) and Ex 26Ri (5′- CCTGCCTTTAAGGCTTCCTT-3′). PCR products were analyzed on 2% agarose gels. Exon 23 skipping was also measured by Taqman quantitative PCR as previously described (21) using Taqman assays designed against the exon 23–24 junction (assay Mm.PT.58.43432707: Forward: 5’-CAGGCCATTCCTCTTTCAGG -3’; reverse: 5’-GAAACTTTCCTCCCAGTTGGT-3’; Probe: 5’-TCAACTTCAGCCATCCATTTCTGTAAGGT-3’) and exon 22–24 junction (Forward: 5’- CTGAATATGAAATAATGGAGGAGAGACTCG-3’; reverse: 5’-CTTCAGCCATCCATTTCTGTAAGGT-3’; Probe: 5’-ATGTGATTCTGTAATTTCC-3’) (Integrated DNA technology). 150 ng of cDNA was used as input per reaction and all assays were carried out in triplicate. Assays were performed under fast cycling conditions on a Biorad CFX384 Touch Real-Time PCR Detection System, and all data were analyzed using the absolute copy number method. For a given sample the copy number of skipped product (exon 22–24 assay) and unskipped product (exon 23–24 assay) were determined using the standards Ex20–26 and Ex20–26Delta23 respectively (gBlocks gene fragments from Integrated DNA technology). Exon 23 skipping was then expressed as a percentage of total dystrophin (calculated by the addition of exon 22–23 and exon 22–24 copy numbers).

### Western blot analysis

Protein lysates were obtained from pooled muscle sections homogenized using the Precellys 24 (Bertin Instruments, France) in RIPA buffer (Thermo Fisher Scientific, USA) complemented with SDS powder (5% final) (Bio-Rad, France) and protease inhibitor cocktail (ThermoFisher Scientific, USA). Protein extracts were denatured at 100°C for 3 min and centrifuged at 13 000 rpm for 10 min at 10°C. Supernatants were collected and the total protein concentration was determined with the BCA Protein Assay Kit (Thermo Fisher Scientific, USA). 25 μg of protein were loaded onto NuPAGE 3–8% Tris-acetate protein gels (Invitrogen), following manufacturer instructions. Dystrophin protein was detected by probing the membrane with NCL-DYS1 primary monoclonal antibody (NCL-DYS1; Novocastra, Newcastle, UK) and vinculin, used as internal control, was detected with the hVin-1 primary antibody (Sigma), followed by incubation with a goat anti-mouse secondary antibody (IRDye 800CW Goat anti-mouse IgG, Li-Cor, Germany). Bands were visualized using the Odyssey CLx system (Li-Cor, Germany) and quantification was done using the Empiria Studio software (Li-Cor, Germany) based on a standard curve specific of each muscle and made of a mix of WT and *mdx* control lysates to obtain defined % of dystrophin.

For Myomesin-3 detection, mouse sera were diluted at 1:20 before loading onto 3–8% Criterion™ XT Tris-Acetate Protein Gel, following manufacturer's instructions (Biorad, France). Myomesin-3 protein was detected by probing the nitrocellulose membrane with MYOM3 primary rabbit polyclonal antibody (MYOM3, Proteintech, Manchester, UK), followed by incubation with a goat anti-rabbit secondary antibody (IRDye 800CW Goat anti-rabbit IgG, Li-Cor, Germany). Bands were visualized using the Odyssey Imaging System (Biosciences, Lincoln, USA). Signals intensity in treated samples were quantified and normalized to PBS control mice signals using the Image Studio software (Li-Cor, Germany).

### Immunohistochemistry analysis

Sections of 10 μm were cut from at least two-thirds of the muscle length of the various tissues (quadriceps, diaphragm, and cardiac muscle) at 100 μm intervals. Cryosections were examined for dystrophin expression using the rabbit polyclonal antibody Dystrophin (dilution 1:500; cat. number RB-9024-P Thermo Scientific), which was then detected by goat anti-rabbit IgGs Alexa 488 (dilution 1:500; Thermo Scientific). Controls prepared by omitting primary antibody showed no specific staining. Images were taken at equivalent locations and exposure times using a Leica DMI-4000 microscope (Leica Microsystems, ×20 objective) equipped with a Zyla 5.5 sCMOS camera (Oxford Instruments Group). Images were cropped and scale bars of 100 μm were added using ImageJ software.

Brain fresh-frozen 30 μm-thick cryosections were collected onto Superfrost+^®^ slides, thawed for 2 min at room temperature (RT), fixed in acetone/methanol (1:1) for 5 min at –20°C, washed in PBS, incubated first in a blocking solution for 45 min (10% normal goat serum, 0.3% Triton X-100 and 1% BSA), then overnight at 4°C with a monoclonal anti-dystrophin primary antibody (DYS1 Leica; dilution: neat) and washed and incubated with secondary antibody Alexa 647 (1:400, 1 h at RT). Controls prepared by omitting the primary antibody showed no specific staining. Images were taken at equivalent locations and exposure times using a laser scanning confocal microscope (Zeiss LSM 700, ×40 objective). Stacks of seven to eight images (1024 × 1024 pixels) spaced by 1 μm were recorded at a magnification of 156 nm/pixel.

### Functional analysis

#### Muscle function

Muscle function of *mdx* mice was evaluated by measuring tibialis anterior muscle contraction *in situ* in response to nerve stimulation as previously described (7). Mice were anesthetized using pentobarbital (60 mg/kg intraperitoneally). Body temperature was maintained at 37°C using radiant heat. The knee and foot were fixed with pins and clamps, and the distal tendon of the muscle was attached to the lever arm of a servo-motor system (305B; Dual-Mode Lever; Aurora Scientific) using a silk ligature. The sciatic nerve was crushed proximally and stimulated distally by a bipolar silver electrode using supramaximal 0.1 ms–duration square-wave pulses. We measured the absolute maximal isometric tetanic force (*P*_0_) generated during isometric contractions in response to electrical stimulation (frequency 75–150 Hz, stimulation train 500 ms). *P*_0_ was determined at *L*_0_ (length at which maximal tension was obtained during the tetanus). Absolute maximal isometric force was normalized to muscle mass as an estimate of specific maximal force (*sP*_0_), that is, specific force.

Fragility was estimated from the force decline resulting from lengthening contraction-induced injury. The sciatic nerve was stimulated for 700 ms (150 Hz stimulation frequency). A maximal isometric contraction of the TA muscle was initiated during the first 500 ms. Then, muscle lengthening (10% *L*_0_) at a velocity of 5.5 mm/s was imposed during the last 200 ms. All isometric contractions were made at an initial length, *L*_0_. Nine lengthening contractions of the TA muscles were performed, each separated by a 60 s rest period. Maximal isometric force was measured 1 min after each lengthening contraction and expressed as a percentage of the initial maximal isometric force. As an indicator of active muscle stiffness, we measured the increase in force during the stretch of the first lengthening contraction. This force was expressed as a percentage of *P*_0_.

#### Respiratory function

The respiratory function of mice was evaluated by whole-body plethysmography using an EMKA technologies plethysmograph, as previously described (6) and essentially as recommended by TREAT-NMD (http://www.treat-nmd.eu/downloads/file/sops/dmd/MDX/DMD_M.2.2.002.pdf).

Briefly, unrestrained conscious mice were placed in calibrated animal chambers and the pressure difference between the reference and animal chambers was measured using a pressure transducer. Mice were allowed to acclimate in the chambers for 45 min at stable temperature and humidity. Data were then collected every 5 s using the iox2 software (version 2.8.0.19; EMKA technologies). Pause and penh were defined and calculated by the following formulas: pause = (TE – RT)/RT and penh = (PEP/PIP) × Pause, where PEP is peak expiratory pressure and PIP is peak inspiratory pressure. The value of each parameter was calculated from an average of 60 recordings of 5 s representing a total of 5 min. Inclusion criteria for each recording were >8 respiration events by 5 s and >80% of success rate as measured by the iox software.

#### Restraint-induced unconditioned fear

Mice were handled firmly but gently using the scruff method, as for standard examination or intraperitoneal injection in laboratory mice. The mouse was restrained by a trained experimenter grasping the scruff and back skin between thumb and index fingers, whilst securing the tail between the third and little fingers and tilting the animal upside-down so that the ventral part of its body faced the experimenter. After 15 s, the mouse was released to a new cage (16 × 28 cm, with 12 cm high walls; illumination: ∼100 lx) and video-tracked for 5 min using the ANY-maze software (Stoelting, USA). All mice were tested between 10:00 am and 1:00 pm. Unconditioned fear responses induced by this acute stress were characterized by periods of tonic immobility (freezing) during the 5 min recording. Complete immobilization of the mouse, except for respiration, was regarded as a freezing response. This was typically quantified as episodes of immobility lasting at least 1s with a 90% immobility sensitivity (10% body motion allowed). In all experiments the percent time spent freezing was calculated for group comparisons. Horizontal (i.e. distance traveled) and vertical activity (number of ups) were also recorded. The investigator was blinded to the group allocations during the experiments.

#### Running test

Run to exhaustion tests were performed using the treadmill LE8710 (Panlab) essentially as recommended by TREAT-NMD (http://www.treat-nmd.eu/downloads/file/sops/dmd/MDX/DMD_M.2.1.003.pdf). Briefly, mice were placed on the belt and the test started at the lowest speed of 5 cm/s to allow a warm-up. Speed was then increased by 1 cm/s every 30 s until exhaustion. Exhaustion was defined as the moment when the mouse would not continue running on the treadmill for 20 s despite gentle nudges to make it do so. At the end of the running test exercise, the total distance run was measured for each mouse.

### Statistical analysis

Data were analyzed with the GraphPad Prism7 software (San Diego, California, USA) and expressed as means ± S.E.M. The ‘n’ refers to the number of mice per group.

Statistical significance was assessed by non-parametric Mann–whitney *U* tests for two-group comparisons. For more than two group comparisons, statistical significance was assessed by one-way ANOVA followed by Dunn's multiple comparisons tests or by two-way ANOVA followed by Tukey's or Sidak's multiple-comparison tests when appropriate.

For correlation analysis between exon-23 skipping and dystrophin restoration levels, a linear regression analysis was performed and statistical significance was assessed using the non-parametric Spearman tests.

ED50 values corresponding to the dose of ASO required to achieve 50% of exon 23 skipping in each tissue were determined by plotting the log dose of ASOs against skipping efficacy (% of exon 23 skipping). The curves obtained were fitted using a four-parameter fit with variable slope and constraining bottom = 0 and top = 1.

Significant levels were set at *P < 0.05, **P < 0.01, ***P < 0.001, ****P < 0.0001.

## RESULTS

### Impact of palmitic acid conjugation on tcDNA-ASO potency

To evaluate the therapeutic potential of palmitic acid conjugation to tcDNA-ASO, we used the previously published tcDNA sequence targeting the donor splice site of the mouse DMD exon 23 (7,17). Palmitic acid was conjugated at the 5’end of tcDNA-ASO containing a full PO backbone (named palm-tcDNA-PO) or a full PS backbone (named palm-tcDNA-PS) using a C6-amino chain as a linker with a phosphorothioate bond (Figure 1A). We first compared the pharmacokinetic properties of these two palm-conjugated tcDNA-ASO to their unconjugated analogues after systemic delivery in adult *mdx* mice, which carry a nonsense mutation in exon 23 of the *DMD* gene (22). Following a single intravenous (IV) injection at 10 μmol/kg of the conjugated tcDNA (palm-tcDNA-PO, palm-tcDNA-PS) and unconjugated tcDNA (tcDNA-PO and tcDNA-PS), serum samples were collected at the indicated time points via typical sparse sampling design (Figure 1B) and ASO were quantified as previously described (17). Using a non-compartmental approach, we evaluated the effect of palmitic acid conjugation and PS modification to plasma and body exposure. PK parameters were calculated using a linear-trapezoidal rule and are summarized in Table 1. Conjugation with palmitic acid increased the area under the plasma concentration-time curve (AUC) of tcDNA by 10-fold, from 276.99 h*μg/ml and 304.62 h*μg/ml for unconjugated tcDNA-PO and tcDNA-PS respectively to 2031.06 h*μg/ml and 3627.69 h*μg/ml for conjugated palm-tcDNA-PO and palm-tcDNA-PS (Figure 1B, Table 1).

**Table 1.** Pharmacokinetics parameters calculated using a non-compartmental approach. Maximum concentration (Cmax), the area under the plasma concentration-time curve (AUC) and the clearance (CL) of each tcDNA compound is each shown.

TcDNA	Cmax (μg/ml)	AUC_0–*t*_ (h*μg/ml)	AUC_0–∞_ (h*μg/ml)	CL (ml/h/kg)
tcDNA-PO	695.12	276.48	276.99	180.51
tcDNA-PS	740.41	297.70	304.62	164.14
Palm-tcDNA-PO	908.4	2029.38	2031.06	24.62
Palm-tcDNA-PS	738.42	3527.72	3627.69	13.79

Next we investigated the accumulation of these different tcDNA compounds in adult *mdx* mice following four intravenous injections at 10 μmol/kg as represented in Figure 1C. Tissue ASO concentration was quantified in several skeletal muscles (tibialis anterior, gastrocnemius, quadriceps, triceps, biceps, diaphragm), heart, spleen, liver and kidney (Figure 1C). Palmitoyl conjugation significantly improved tcDNA-ASO accumulation of both PO and PS compounds in skeletal muscles (6-fold on average for palm-tcDNA-PS and 28-fold for palm-tcDNA-PO, Supplementary Table S1) relative to unconjugated ASO (*P*-value < 0.0001). Palm-tcDNA-PS was the compound leading to the highest accumulation of ASO in tissues, however the contribution of the palmitoyl was even more remarkable for the PO compound showing a higher fold change compared to unconjugated tcDNA-PO which is otherwise very poorly distributed. Palm-tcDNA-PO also induce significantly higher levels of ASO in tissues than our previously described compound tcDNA-PS (*P*-value < 0.005). Delivery to the spleen, liver and kidney was also improved by the addition of the palmitoyl moiety, leading to nearly similar amount of ASO in kidney with both conjugated ASO (Figure 1C, Supplementary Table S1).

To determine the metabolic fate of the palmitoyl conjugated-ASO in tissues, ASO and metabolites were also extracted and identified by Liquid-Chromatography-Mass-Spectrometry (LCMS) as previously described (6). LCMS quantifications revealed very similar amounts of ASO in tissues than the hybridization-based assay (Supplementary Figure S1), except in the kidney where amounts appeared lower in LCMS. Interestingly, no intact conjugated ASO was detected and the main metabolite isolated in all tissues was the C6-amino-tcDNA (PS or PO), suggesting that the palmitoyl moiety was cleaved from the oligonucleotide at the amide bond.

To determine whether the higher uptake observed with palmitoyl conjugates leads to improved potency, we next evaluated the levels of exon 23 skipping in the various tissues. The RT-PCR results revealed a dystrophin transcript lacking exon 23 for the unconjugated (tcDNA-PS) and conjugated tcDNA compounds in all investigated muscles from tcDNA treated mice (Figure 1D, right panel). Quantification by RT-qPCR confirmed the superiority of palmitoyl conjugated tcDNA-ASOs in all examined tissues, reaching up to 25% of exon 23 skipping in skeletal muscle after only four injections at 10 μmol/kg for the palm-tcDNA-PS. The addition of palmitic acid significantly improved the efficacy of the tcDNA-PS (5-fold on average across the different muscles, Supplementary Table S2) and even more of the tcDNA-PO with an average of 50-fold improvement between the palm-tcDNA-PO and the unconjugated tcDNA-PO (Figure 1D and Supplementary Table S2). The Palm-tcDNA-PS induced higher level of exon 23 skipping (*P*-value = 0.03) than the palm-tcDNA-PO except in diaphragm and heart where levels were similar for both conjugated compounds.

This improved efficacy of the conjugated tcDNA–ASOs correlated with the increase in ASO exposure in tissues and confirmed the potency of palmitoyl conjugation.

### Evaluation of a 12-week treatment with palm-tcDNA at different doses

**Figure 2.**
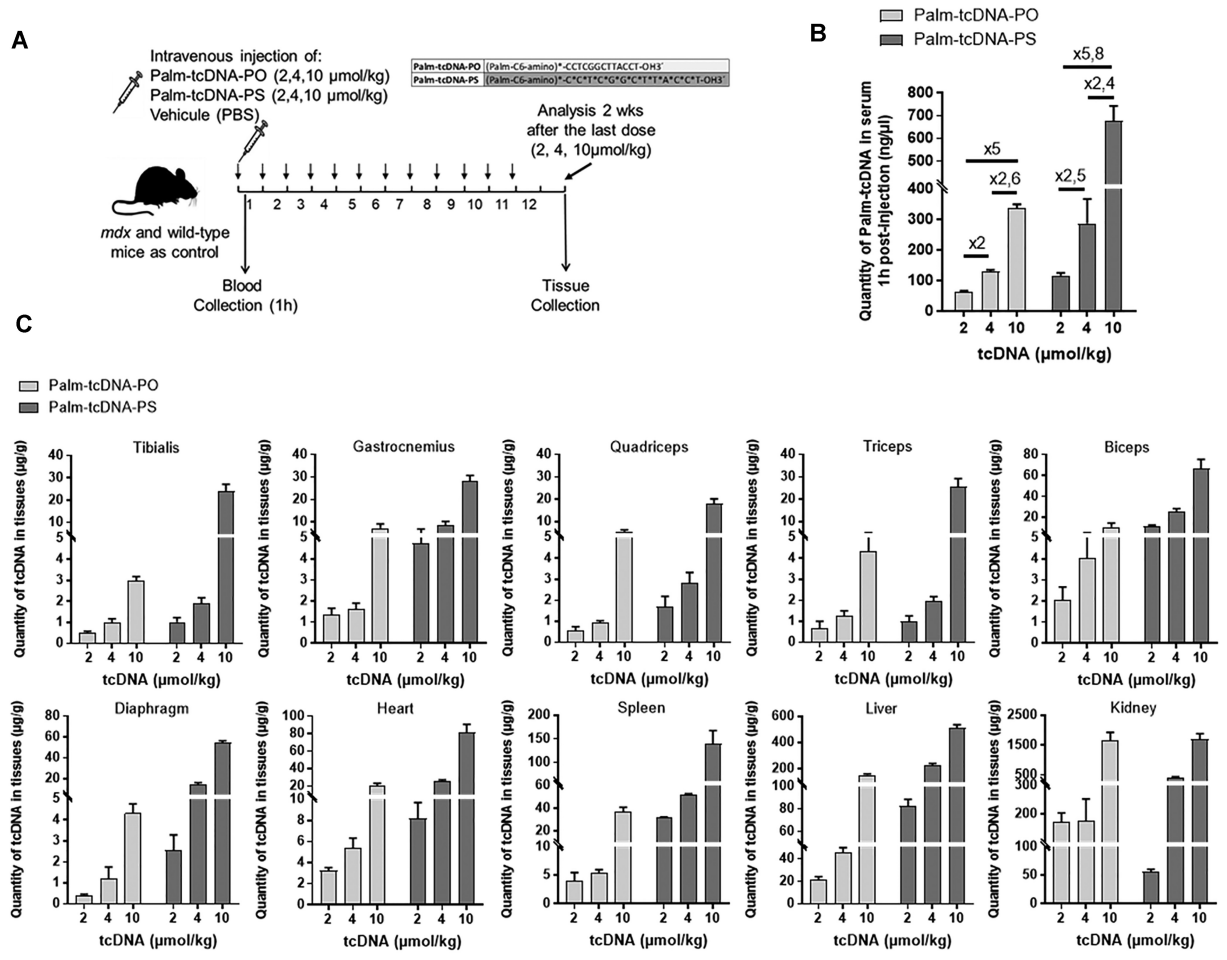
Dose dependant distribution of palmitic acid conjugated tcDNA in tissues following 12-week repeated dosing. (**A**) Schematic representation of the 12-week study protocol with different doses of palmitic acid conjugated tcDNA-ASO. *Mdx* mice were injected intravenously with 2, 4 or 10 μmol/kg/week of conjugated tcDNA (palm-tcDNA-PO or palm-tcDNA-PS) for 12 weeks and euthanized 2 weeks after the last dose for tissue collection. 2, 4 or 10 μmol/kg correspond to approximately 10, 20 and 50 mg/kg. (**B**) Quantification of palm-tcDNA in mouse serum collected 1 h post-injection by fluorescent hybridization assay (*n* = 4 per group; data are represented as mean ± SEM and ratio between the different doses for each compound are shown). (**C**) Quantification of tcDNA-ASO content in various tissues 2 weeks after the end of the 12-week dosing regimen by fluorescent hybridization assay. Results are expressed as mean ± SEM; *n* = 4 mice per group.

We next investigated the therapeutic potential of tcDNA conjugated to palmitic acid following long term treatment at different doses. Adult *mdx* mice were injected intravenously with 2, 4 or 10 μmol/kg/week of palm-tcDNA-PO or palm-tcDNA-PS for 12 weeks (Figure 2A). Serum samples collected 1h after administration confirmed the longer half-life of palm-tcDNA-PS previously observed since we detected higher levels (approximately 2-fold) of palm-tcDNA-PS circulating than palm-tcDNA-PO at all tested doses (Figure 2B). The amount of tcDNA in the serum 1 h post-injection was proportional to the injected doses for both compounds, with calculated ratio very close to the expected ratio of 2, 2.5 and 5 between the 2, 4 and 10 μmol/kg dosing regimen as shown in Figure 2B. ASO tissue concentrations were measured in the various tissues (tibialis anterior, gastrocnemius, quadriceps, triceps, biceps, diaphragm, heart, spleen, liver and kidney) 2 weeks after the end of the 12-week dosing regimen. We observed a dose dependant accumulation of ASOs in tissues with the highest amount observed with 10μmol/kg treated samples for both palm-tcDNA-PO and -PS. Significantly higher levels of ASO (∼4-fold) were found in tissues from mice treated with palm-tcDNA-PS than palm-tcDNA-PO at 4 and 10 μmol/kg (*P*-values = 0.03 and <0.0001 respectively) except in kidneys where the amount of the palm-tcDNA was similar between the two conjugated compounds. Remarkably, we were able to quantify the palm-tcDNA even at the low dose of 2 μmol/kg, which reflects the amelioration of tissue uptake by the palmitoyl conjugation. When comparing the ASO concentrations found in tissues following 12 weeks of treatment with those measured after 4 weeks of treatment at 10 μmol/kg (Supplementary Figure S2A), we found ∼2 times higher levels in palm-tcDNA-PS treated tissues. Interestingly, levels were quite similar (1.3 fold) in the palm-tcDNA-PO treated samples between 4 and 12 weeks of treatment, suggesting a higher clearance rate of this compound in tissues.

**Figure 3.**
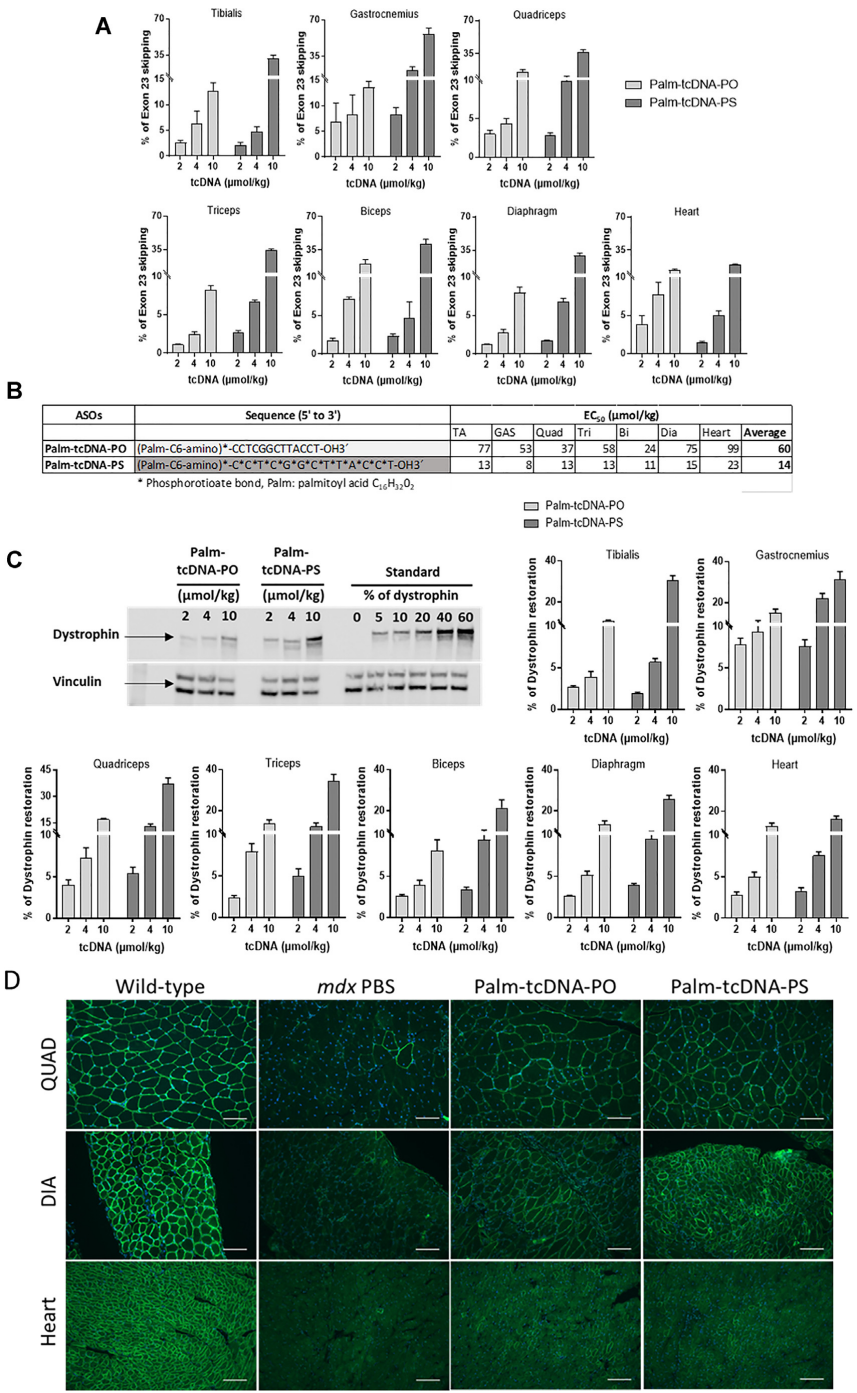
Skipping efficacy and dystrophin rescue following 12-week repeated dosing of palm-tcDNA-ASO. (**A**) Quantification of exon 23 skipping levels in different muscle tissues 2-week after the end of the 12-week dosing regimen (palm-tcDNA-PO or palm-tcDNA-PS at 2, 4 or 10 μmol/kg/week) by RT-qPCR. Results are expressed as mean ± SEM; *n* = 4 mice/group. (**B**) ED50 values corresponding to the dose of ASO required to achieve 50% of exon 23 skipping in each tissue were determined with GraphPad Prism 7 software for palm-tcDNA-PO or palm-tcDNA-PS. (**C**) Detection and quantification of dystrophin restoration by western blot analysis in the different muscle tissues 2 weeks after the end of the 12-week dosing regimen (palm-tcDNA-PO or palm-tcDNA-PS at 2, 4 or 10 μmol/kg/week). The blot shows a representative example of dystrophin restoration in the quadriceps of one of the four animals per group. Results are expressed as mean ± SEM; *n* = 4 mice/group. (**D**) Detection of the dystrophin protein (green staining) by immunostaining on transverse sections of muscle tissues (quadriceps, diaphragm and heart) from WT and *mdx* mice treated with PBS or palm-tcDNA-PO or palm-tcDNA-PS for 12 weeks at 10 μmol/kg/week. Nuclei are labelled in blue (DAPI). Scale bar, 100 μm.

The efficacy of the 12-wk treatment with conjugated tcDNAs (palm-tcDNA-PO and palm-tcDNA-PS) was then evaluated by RT-qPCR for the different dosing regimen (2, 4 and 10 μmol/kg). Both Palm-tcDNA compounds induced exon 23 skipping in skeletal muscles in a dose dependent manner. We detected levels from 1 to 8% for the low dose of 2 μmol/kg, from 2 to 21% for the mid dose of 4 μmol/kg and levels from 8 to 56% for the 10 μmol/kg dose (Figure 3A). Calculation of the ED50 indicated a 4-fold difference between the two compounds in favour of the palm-tcDNA-PS across all muscles (Figure 3B). However, the palm-tcDNA-PS induced only statistically significant higher levels of exon skipping at the dose of 10μmol/kg, whereas levels were quite similar between the two compounds at 2 and 4 μmol/kg (*P*-values > 0.99 and 0.91, respectively). When comparing skipping levels obtained after 4 or 12 weeks of injections (Supplementary Figure S2B), we found very similar ratio for palm-tcDNA-PO and palm-tcDNA-PS (2.4 and 2.5) suggesting that efficacy increases in the same way for both compounds despite the higher clearance of palm-cDNA-PO compound.

Based on biodistribution data and efficacy levels, we calculated the ratio of the exon skipping levels on ASO quantity per tissue in order to evaluate the therapeutic potential of each compound, i.e. determine which compound produces the highest levels of exon skipping with minimal tcDNA accumulation. The palm-tcDNA-PO consistently displays higher ratio than the palm-PS (mean ratio of 2.5 ± 0.3 across all muscles tissues and dosing regimen compared to 1.7 ± 0.2 for the palm-tcDNA-PS) (Supplementary Table S3).

We then quantified dystrophin restoration by western blot in the different muscle tissues following treatment with both palm-tcDNA (PO or PS) at 2, 4 and 10 μmol/kg for 12 weeks. Levels of dystrophin restoration correlated with the levels of exon skipping (Spearman correlation test, *R* = 0.89, *P* < 0.0001) at the different dosing regimen. The amounts of dystrophin protein found in skeletal muscle tissues were significantly different between the two compounds at the 10 μmol/kg dose (*P* < 0.0001), ranging from 8 to 17% with palm-tcDNA-PO and from 21% to 37% with palm-tcDNA-PS after the 12 weeks of treatment (Figure 3C). Levels of dystrophin protein in the heart reached 12% and 16% for the palm-tcDNA-PO and palm-tcDNA-PS respectively (not statistically different between the two compounds), demonstrating the ability of the palmitoyl-tcDNA compounds to efficiently restore dystrophin in the cardiac muscle. We also compared the levels of dystrophin expression after 4 or 12 weeks of treatment at the dose of 10 μmol/kg with both compounds and found a proportional ∼3-fold increase to the number of injections, confirming the accumulation of the protein over the period of treatment (Supplementary Figure S2C). Immunostainings were performed on quadriceps, diaphragm and heart muscle sections and revealed the correct expression and localization of dystrophin protein at the sarcolemma level of muscle fibers after the palm-tcDNA (PO and PS) treatment (Figure 3D).

### Functional evaluation of conjugated tcDNA after 12 weeks of treatment

**Figure 4.**
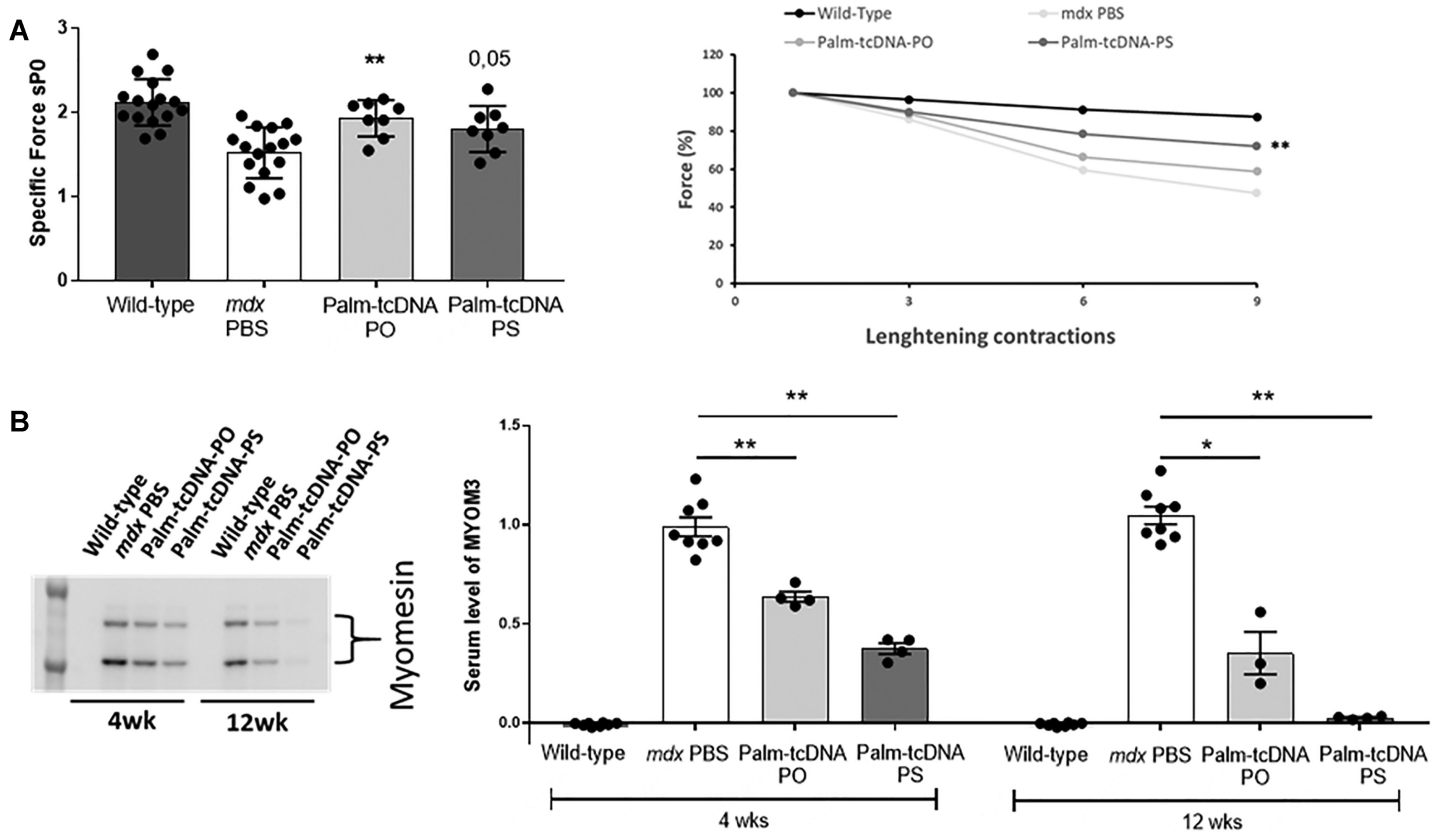
Functional evaluation following 12-week repeated dosing of palm-tcDNA-ASO treatment. (**A**) Maximal specific force (sP0) (left panel) and percentage of force drop following a series of eccentric contractions (right panel) measured on semi-isolated tibialis anterior (TA) muscles from *mdx* mice treated with palm-tcDNA-PO or palm-tcDNA-PS (*n* = 4 mice/group and 2 TA analysed per mouse) for 12 weeks at 10 μmol/kg/week and compared to WT and PBS control *mdx* mice (*n* = 8 mice/group). Results are expressed as mean ± SEM. **P < 0.01 compared to PBS treated controls (Mann–Whitney *U* tests). (**B**) Detection and quantification of MYOM3 levels by western blot in serum of *mdx* mice treated with palm-tcDNA-PO or palm-tcDNA-PS (*n* = 4 mice/group) for 4 or 12 weeks at 10 μmol/kg/week and compared to WT and PBS control *mdx* mice (*n* = 8 mice/group). Results are expressed as mean ± SEM. *P < 0.05 and **P < 0.01 compared to PBS treated controls (Mann–Whitney *U* tests).

In order to evaluate the functionality of the restored dystrophin, functional properties were then investigated in palm-tcDNA treated mice at 10 μmol/kg. We first assessed the maximal specific force and resistance to eccentric contraction-induced skeletal muscle injury, which reflects on the structural integrity of the muscle fibers. *Tibialis anterior* (TA) muscles from control *mdx* mice showed a decrease of 28% in maximal specific force compared to the wild-type muscle (Figure 4A). In contrast, treatment with palm-tcDNA-PO and palm-tcDNA-PS inducing 10 and 30% of dystrophin expression respectively, improved the maximal specific force compared to control *mdx* mice (*P* = 0.001 and *P* = 0.05 for palm-tcDNA-PO and PS, respectively). Moreover, TA muscles from control *mdx* mice were unable to sustain tetanic tension, falling to 47% of their initial force after nine eccentric contractions (Figure 4A, right panel). Palm-tcDNA treatment improved the resistance to tetanic contractions since TA muscles treated with palm-tcDNA-PO and palm-tcDNA-PS maintained 58% and 71% respectively of their force following the eccentric contractions, which correlated with the observed levels of protein restoration. Respiratory function was also explored in *mdx* mice following treatment using whole-body plethysmography. We first confirmed that *mdx* control mice showed abnormalities for some respiratory parameters, such as the enhanced pause (Penh) and the peak expiratory flow (PEF) compared to age-matched wild-type mice (Supplementary Figure S3A). Both parameters appeared slightly improved after palm-tcDNA (PO and PS) treatment at 10 μmol/kg although not statistically significantly. The functionality of the restored dystrophin was further explored using a treadmill test in order to evaluate the ability of treated *mdx* mice to sustain a continuous effort during a long period of time. We observed a drastic amelioration of the endurance capacity of the palm-tcDNA-PS treated mice after 6 weeks or 12 weeks of treatment (*P*-value = 0.0002 and < 0.0001 respectively). The treated mice even reached the wild-type mice performance (Supplementary Figure S3B).

It has been previously demonstrated that two fragments of the myofibrillar structural protein myomesin-3 (MYOM3) are abnormally present in sera of Duchenne Muscular Dystrophy (DMD) patients as well as animal models of DMD including the *mdx* mouse model (23). The levels of MYOM3 fragments in serum can be used to evaluate the efficacy of a treatment as it has already been considered a useful therapy-responsive biomarker in animal models as well as in patients. We therefore evaluated the level of MYOM3 in serum of palm-tcDNA-PO or -PS treated mice after 4 and 12 weeks of treatment. Levels of MYOM3 in the serum following treatment with the palm-tcDNA-PO were significantly dropped by 37% and 65% (*P*-value = 0.002 and 0.009) after 4 and 12 weeks of treatment (Figure 4B). MYOM3 levels were even more strongly reduced with the palm-tcDNA-PS where we found a total reduction (99,9%) after the 12-wk treatment (*P*-value = 0.0002) (Figure 4B). These results confirm the therapeutic benefit of the treatment with the palmitoyl-tcDNA oligonucleotide.

### Effect of palm-tcDNA in the CNS

**Figure 5.**
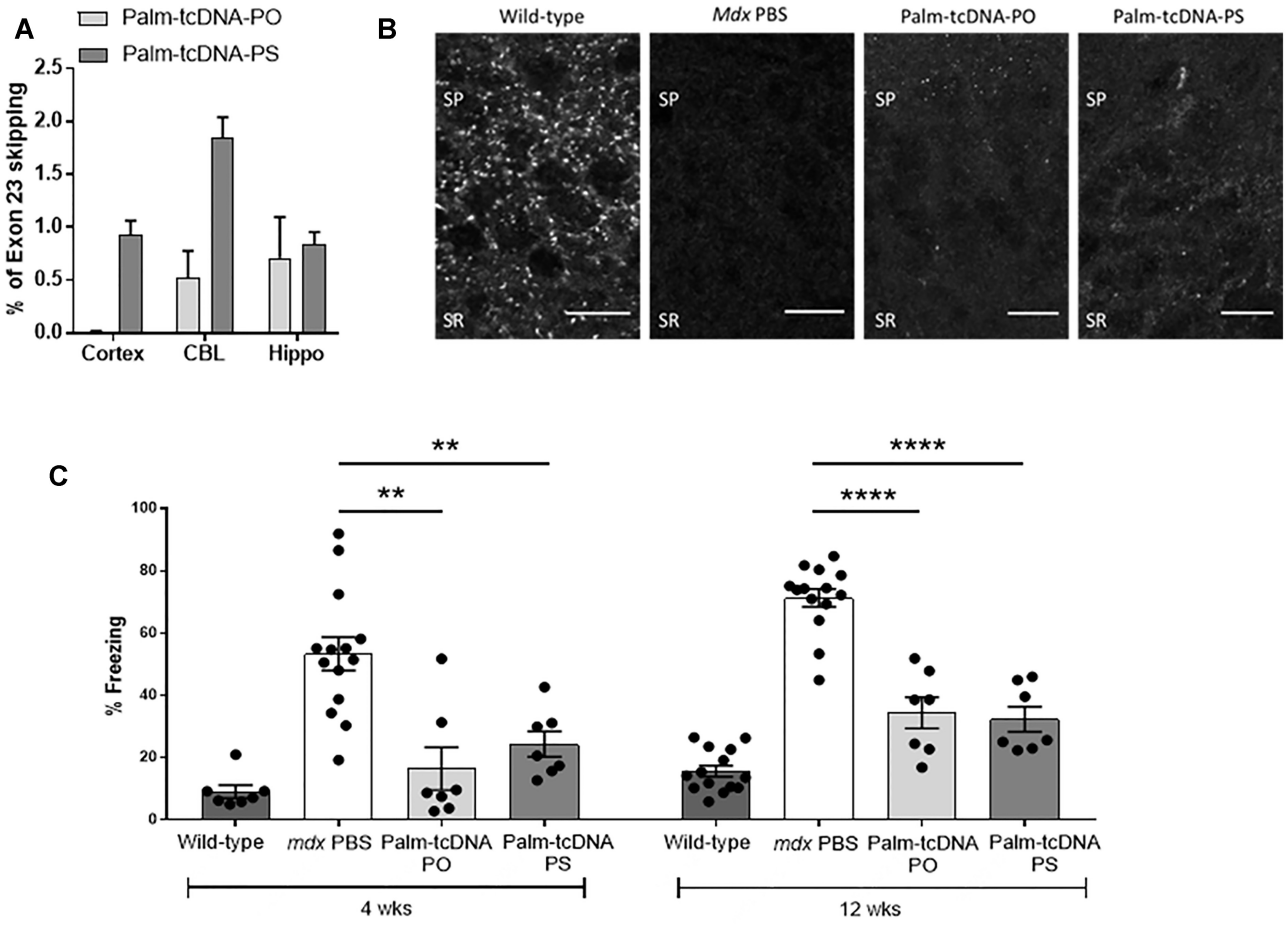
Palm-tcDNA-ASO effect on central nervous system in treated *mdx* mice. (**A**) Quantification of exon 23 skipping by qRT-PCR in the cortex, cerebellum (CBL), and hippocampus (Hippo) following intravenous injection of 10 μmol/kg/week of palm-tcDNA-PO or palm-tcDNA-PS for 12 weeks. Results are expressed as mean ± SEM; *n* = 4 mice per group. (**B**) Detection of restored dystrophin by immunostaining in the stratum pyramidale (SP) and proximal stratum radiatum (SR) of the CA1 hippocampus in wild-type and *mdx* mice treated with PBS, palm-tcDNA-PO or palm-tcDNA-PS (10 μmol/kg/week). Scale bar 12 μm. (**C**) Restraint-induced unconditioned fear responses expressed as a percentage of freezing time in wild-type and *mdx* mice treated with PBS, palm-tcDNA-PO or palm-tcDNA-PS (10 μmol/kg/week) after 4 weeks and 12 weeks of treatment. **P < 0.01 and ****P < 0.0001 compared to *mdx* controls (Mann–Whitney *U* tests); Results are expressed as mean ± SEM; *n* = 7 per palm-tcDNA treated group and *n* = 14 for wild-type and *mdx* PBS control (mice from the wash-out groups were included in this non-invasive test).

We previously described that tricyclo-DNA ASOs are able to cross the blood-brain barrier (BBB) and promote exon 23 skipping at the mRNA level after systemic delivery (6,7). We therefore investigated the effect of the palmitic-acid conjugation on this unique property of the tcDNA. Following a 12-week systemic treatment with palm-tcDNA (-PO or -PS) at 10 μmol/kg we quantified by RT-qPCR the levels of exon 23 skipping in cortex, cerebellum and hippocampus of treated *mdx* mice and confirmed the capacity of the conjugated palm-tcDNA to induce low levels of exon 23 skipping in the CNS, with no significant differences between the two conjugated compounds (-PO or -PS) (Figure 5A). We also observed a partially restored expression of the dystrophin protein with correct localization in stratum pyramidal (SP) and proximal stratum radiatum (SR) of CA1 hippocampus by immunostaining following the treatment with the conjugated tcDNA (Figure 5B).

The loss of dystrophin in the *mdx* mouse model of DMD has been associated with cognitive and emotional alterations, and an enhanced defensive behavior in response to a mild stress has been reported as a main phenotype (24,25). In order to evaluate the beneficial impact of treatment with the conjugated palm-tcDNA (-PO or -PS) on this emotional behavior, we measured the duration of tonic immobility (freezing) that resulted from a restraint-induced fear response in *mdx* mice at two different time points (after 4 and 12 weeks of treatment) (Figure 5C). The mice were observed for a period of 5 min following this acute stress and as expected control *mdx* mice spent about 70% of the time freezing, in contrast to only 11% for the WT mice. In line with the detected exon skipping and rescued dystrophin expression in the brain following treatment with both conjugated palm-tcDNAs, we measured a significant improvement of the *mdx* emotional phenotype, reflected by a decreased freezing time in the palm-tcDNA-PO and palm-tcDNA-PS treated mice during the 5 minutes testing period after only 4 weeks (*P*-value = 0.001 after 4 weeks of treatment and *P*-value < 0.0001 after 12 weeks of treatment) (Figure 5C).

### Persistence of treatment effect after a 12-week recovery period

**Figure 6.**
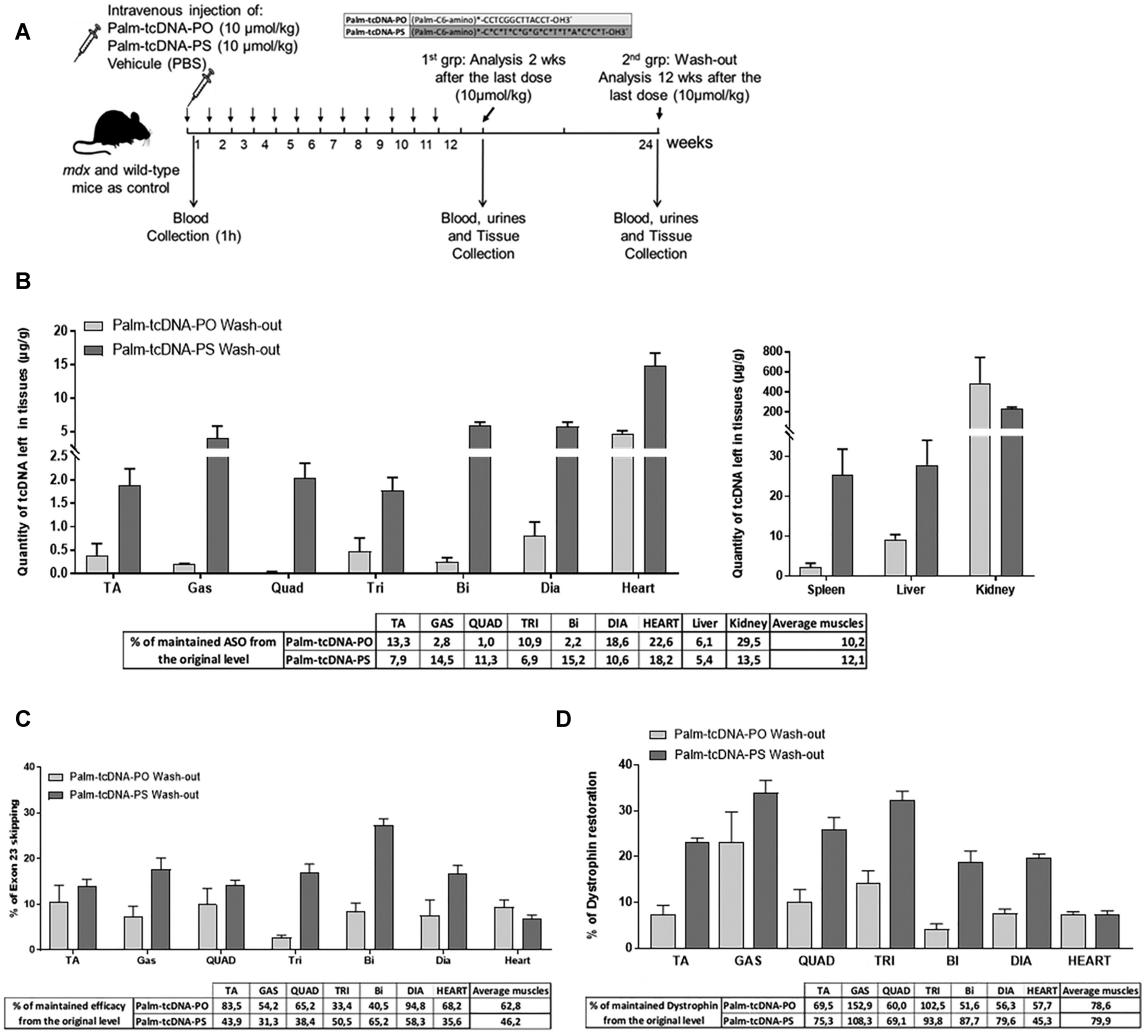
Palm-tcDNA-ASO efficacy is maintained after 12 weeks of wash-out period. (**A**) Schematic representation of the 12-week study protocol at 10 μmol/kg/week of palm-tcDNA-PO or palm-tcDNA-PS followed by a 12-week wash-out period. (**B**) Quantification of tcDNA compounds in the different tissues after the 12-week wash-out period by fluorescent hybridization assay. Results are expressed as mean ± SEM; *n* = 5 for palm-tcDNA-PO and *n* = 3 for palm-tcDNA-PO. The percentage of remaining ASO compared to the content measured 2 weeks after the last dose is indicated below the graphs. (**C**) Quantification of exon 23 skipping levels in different muscle tissues by taqman RT-qPCR after the 12-week wash-out period. Results are expressed as mean ± SEM; *n* = 3 for palm-tcDNA-PO and *n* = 5 for palm-tcDNA-PS. The percentage of remaining exon 23 skipping levels compared to the those measured 2 weeks after the last dose is indicated below the graphs. (**D**) Quantification of dystrophin restoration by western blot in the different muscle tissues after the 12-week wash-out period. Results are expressed as mean ± SEM; *n* = 3 for palm-tcDNA-PO and *n* = 5 for palm-tcDNA-PS. The percentage of remaining dystrophin expression compared to the levels measured 2 weeks after the last dose is indicated below the graphs.

Because treatment of DMD patients involves a lifetime medication, it is important to evaluate periods of wash-out to avoid the potential toxicity due to ASO accumulation in tissues like liver, kidney and spleen, without losing the benefit of the treatment. For this purpose, we investigated the persistence of the 12-week treatment effects (with both -PO and -PS palm-tcDNA) after a recovery period of 12 weeks (named the wash-out period) (Figure 6A). Following the wash-out period, tcDNA compounds were still detected in all the investigated tissues at ∼10–12% of the originally detected levels, demonstrating that ∼90% of ASO have been cleared (Figure 6B). Higher levels were detected for the palm-tcDNA-PS compared to the palm-tcDNA-PO except in the kidney. Interestingly, when measuring the levels of exon 23 skipping (Figure 6C), we found on average 62 and 46% of the originally detected levels after palm-PO and palm-PS treatment respectively. We also observed the persistence of dystrophin protein across the different skeletal muscles with an average of ∼80% maintained dystrophin across the tissues for both conjugated compounds (Figure 6D). We then evaluated the functional parameters and found that improvements in force drop, specific force and freezing response were also maintained after the recovery period for both conjugated palm-tcDNAs (Supplementary Figure S4). The MYOM3 levels were still significantly decreased in the serum of *mdx* mice treated with palm-tcDNA-PO and palm-tcDNA-PS after 4 weeks of recovery period (*P*-value = 0.01) but only the mice treated with the palm-tcDNA-PS maintained this significance after the 12 weeks of wash-out (Supplementary Figure S4B).

### Safety profile of conjugated palm-tcDNA

**Figure 7.**
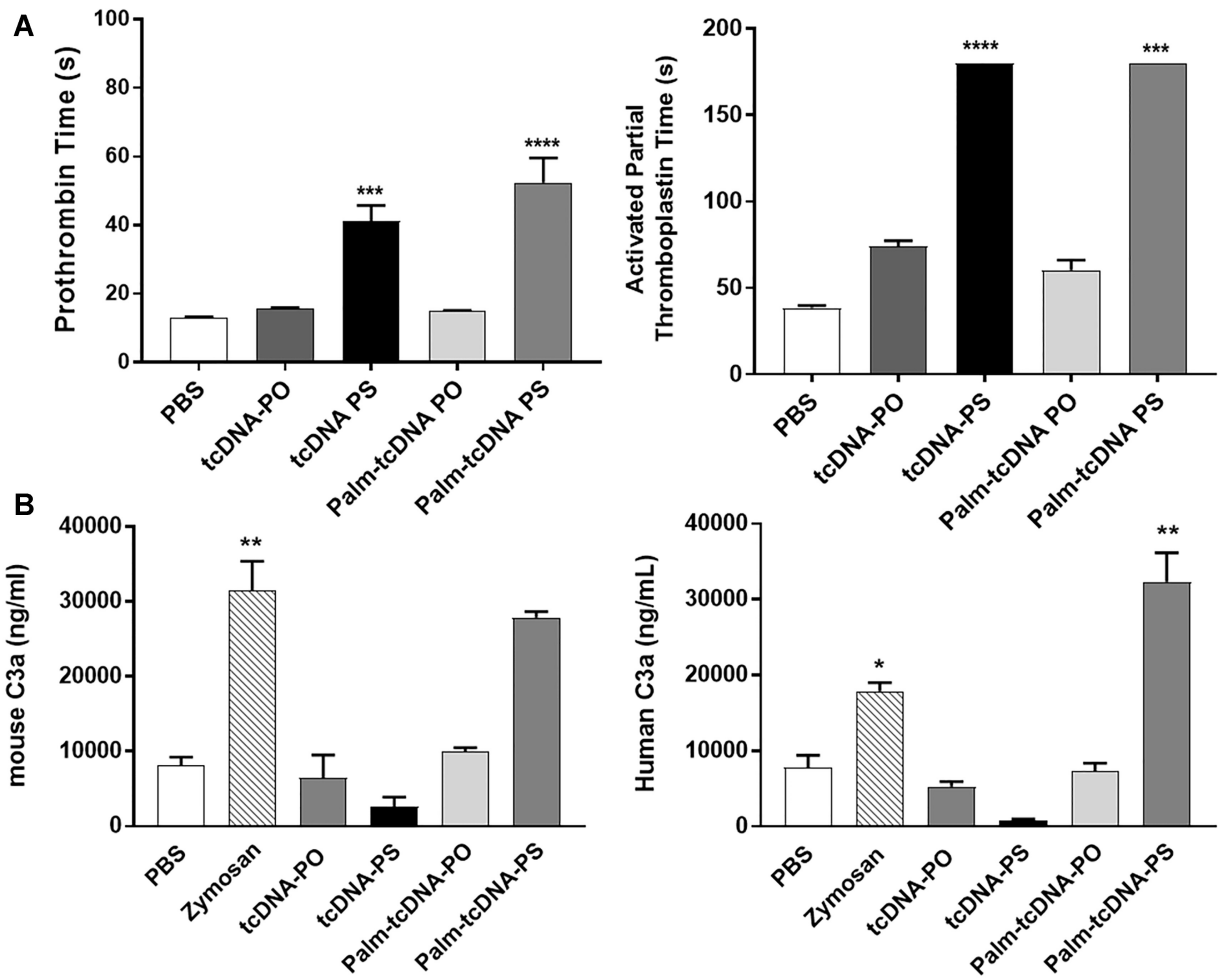
*In vitro* evaluation of the palm-tcDNA-ASO on clotting times and complement activation. (**A**) To determine the effect of the different tcDNA-ASO on coagulation pathways, the prothrombin time (PT) (left) and the activated partial tromboplastin (aPTT) (right) were analysed in human plasma incubated with PBS (*n* = 12), tcDNA-PO (*n* = 6), tcDNA-PS (*n* = 6), palm-tcDNA-PO (*n* = 5) or palm-tcDNA-PS (*n* = 5). Results are expressed as mean ± SEM. ****P < 0.0001 compared to PBS (one-way ANOVA). (**B**) Mouse and human C3a anaphylotoxin were analysed by ELISA in mouse and human serum samples incubated with tcDNA-PO, tcDNA-PS, palm-tcDNA-PO or palm-tcDNA-PS. PBS and Zymosan were used as negative and positive control respectively. Results are expressed as mean ± SEM; *n* = 5 per tcDNA treated group and *n* = 12 for PBS and Zymosan controls. * *P* < 0.05, ** *P* < 0.01, ***P < 0.001, ****P < 0.0001 compared to PBS.

Safety pharmacological evaluation is a crucial aspect in drug development that needs to be considered relatively early for every new molecule with therapeutic purposes. PS-ASOs are known to influence the coagulation cascade and the alternative pathway of the complement system as a protein binding related effect (11,12). We therefore evaluated *in vitro* the intrinsic (activated partial thromboplastin time or aPTT) and extrinsic (prothrombin time or PT) coagulation pathways in mouse plasma as well as complement activation in mouse and human serum samples. Appropriate plasma or serum samples were incubated with 2 mg/ml of each tcDNA-ASO. As expected, the results showed no effect of the palm-tcDNA-PO (and tcDNA-PO) on coagulation times as opposed to PS-containing tcDNA which significantly increase PT and aPTT (Figure 7A). Similarly, tcDNA-PO compounds had no effect on complement activation in mouse or human serum, whereas a significant increase in C3a split product was detected *in vitro* in human serum samples incubated with palm-tcDNA-PS (Figure 7B). This effect was found dose dependent as lower doses did not induce significant increase in C3a (Supplementary Figure S5A) and correlated with *in vivo* findings where no complement activation was measured after administration of 10 μmol/kg of either palm-tcDNA (Supplementary Figure S5B).

**Figure 8.**
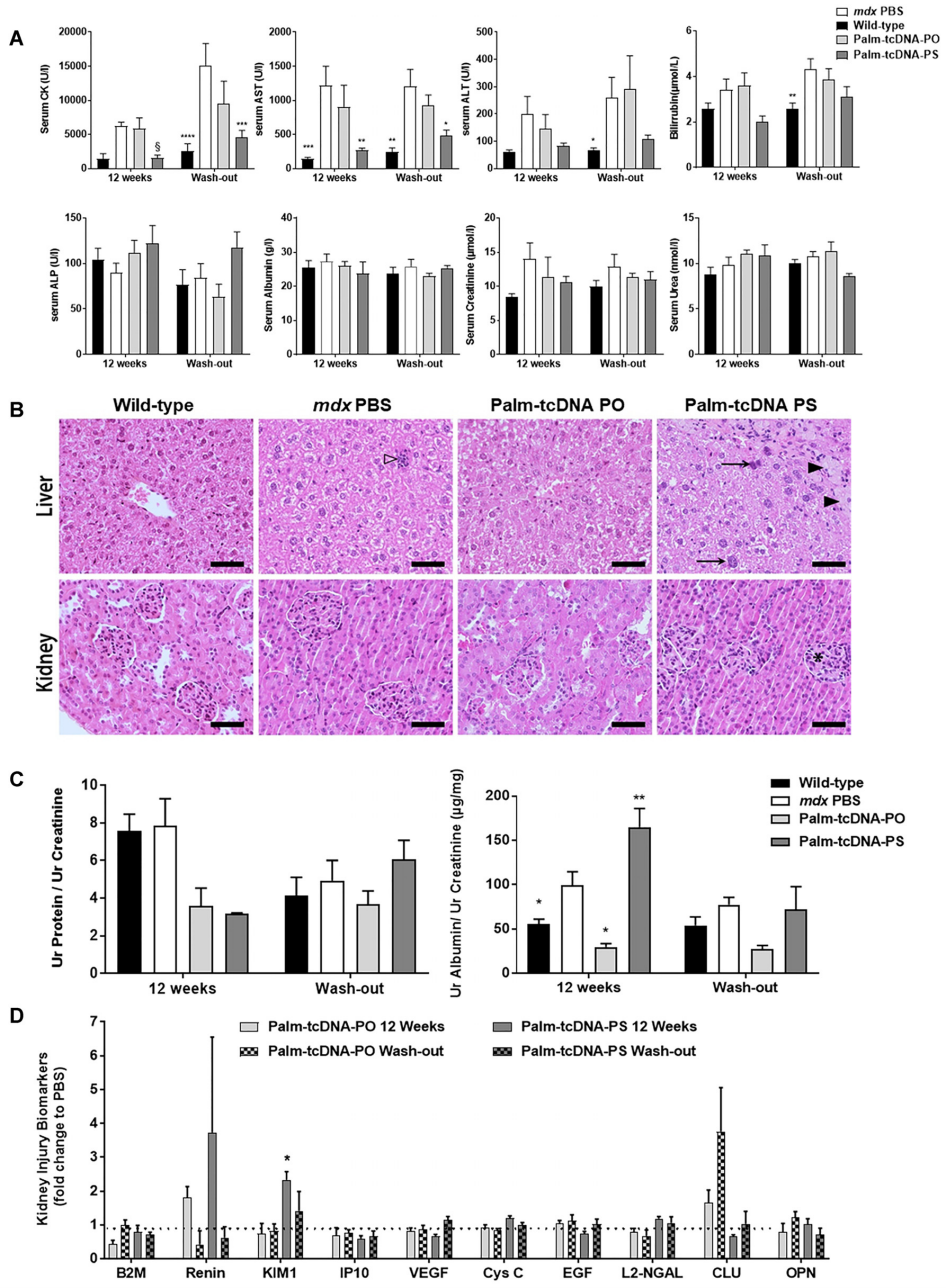
Evaluation of palm-tcDNA-ASO safety profile. (**A**) Serum CK, AST, ALT, bilirubin, ALP, albumin, creatinine and urea levels were measured at the end of the 12-wk treatment (12 weeks) or after a 12-week washout period (wash-out) in wild-type (*n* = 7) and *mdx* mice treated with PBS (*n* = 7), palm-tcDNA-PO (*n* = 3) or palm-tcDNA-PS (*n* = 5). Results are expressed as mean ± SEM. * *P* < 0.05, ** *P* < 0.01, ***P < 0.001, ****P < 0.0001 compared to PBS (two-way ANOVA). § *P* = 0.0025 compared to PBS (Mann–Whitney *U* tests). (**B**) Histological presentation of wild-type mice and *mdx* mice treated with PBS, palm-tcDNA-PO or palm-tcDNA-PS at 10 μmol/kg/wk for 12 weeks. In liver (upper panel), small foci of inflammatory cell infiltration (open arrowhead) were scattered in the liver parenchyma of *mdx* PBS mice compared to WT mice in which no such focus were observed. No additional lesions were present in *mdx* mice after palm-tcDNA-PO treatment. In palm-tcDNA-PS mice, foci of hepatocytes with intracytoplasmic vacuolization and fainted eosinophilic content (black arrowhead) were observed. Additionally, an increase in size heterogeneity of hepatocyte nuclei was present with notably numerous binucleated cells and meganucleation (arrow). In kidney (lower panel), no lesions were present in *mdx* mice after PBS or palm-tcDNA-PO treatment compared to WT mice. A slight increase of cellular density (*) in glomeruli was sometimes noted in mice treated with palm-tcDNA-PS. Hemalun-Eosin-Saffron staining. Scale bar = 50 μm. (**C**) Total protein and albumin levels in the urine of wild-type (*n* = 7) and *mdx* mice treated with PBS (*n* = 7), palm-tcDNA-PO (*n* = 3) or palm-tcDNA-PS (*n* = 5) at 10 μmol/kg/week for 12 weeks. Urines were collected at the end of the 12-week treatment (12 weeks) or after a 12-week washout period (wash-out). Results are normalised to creatinine levels and expressed as mean ± SEM. * *P* < 0.05 compared to PBS (two-way ANOVA). (**D**) Kidney injury biomarkers (KIB) were evaluated in urines collected from *mdx* mice treated with palm-tcDNA-PO (*n* = 3) or palm-tcDNA-PS (*n* = 5) at 10 μmol/kg/week for 12 weeks. Urines were collected at the end of the 12-week treatment (12 weeks) or after a 12-week washout period (wash-out). Results are normalised to *mdx* PBS levels and expressed as mean ± SEM; *P < 0.05 compared to PBS (Mann–Whitney *U* tests).

Additional adverse and toxic effects of antisense molecules may include liver or renal injury, especially in repeated-dose studies considering the amount of ASO which end up in liver and kidney. In this regard, we first analyzed the serum levels of various general biomarkers in mice following 12 weeks of treatment with 10 μmol/kg/wk of palm-tcDNA-ASOs either 2 weeks after the last dose or after a 12-week wash-out period. The serum creatine kinase (CK) level, a marker for muscle injury, was largely reduced in mice treated with palm-tcDNA-PS for 12 weeks and this was also observed for AST levels reflecting the efficacy of the treatment. Interestingly this effect was still detected after the 12-week wash-out period. Apart from these positive effects resulting from the improved dystrophic pathology of treated *mdx* mice, we observed no significant difference in serum levels of transaminases (ALT and AST), bilirubin, alkaline phosphatase (ALP), albumin, creatinine and urea of palm-tcDNA treated mice compared to *mdx* control mice (Figure 8A).

Additionally, we explored the histopathological profile of liver and kidney in treated *mdx* mice in order to determine and evaluate the potential toxic response within the tissues. In the liver, most treated animals (3/4 for palm-tcDNA-PO and 4/5 for palm-tcDNA-PS) displayed some small scattered foci of inflammatory cell infiltration centered around a few individual necrotic hepatocytes (Figure 8B). On the contrary, only few (2/6) control *mdx* animals displayed similar lesions that are commonly reported in adult rodents. Similar findings were observed after the wash-out period (Supplementary Figure S5C). Additionally, in all palm-tcDNA-PS treated animals, hepatocytic nuclei displayed an increased degree of pleomorphism corresponding to irregular nucleus size (anisokaryosis), heterogeneous aspect of chromatin and in most severe cases, atypias like binucleation, meganucleation or meganucleolation. This was only observed in one mouse treated with the palm-tcDNA-PO. The intensity of pleomorphism was scored and showed significant difference between the 2 treatments with a score of 2.4 ± 0.6 for palm-tcDNA-PS and 0.25 ± 0.5 for palm-tcDNA-PO (significant difference, Mann-Whitney, *P* < 0.05). Of note, a null score was attributed to all control animals. After 12 weeks of wash-out, pleomorphism was only present in animals having received palm-tcDNA-PS with a score of 1.2 ± 0.8. Also, in palm-tcDNA-PS treated animals exclusively, clusters of hepatocytes with macrovesicular intracytoplasmic vacuoles with an eosinophilic content were observed. In large clusters, individual hepatocytic necrosis was often associated with a mild infiltration of mixed inflammatory cells. This lesion was observed in all animals under palm-tcDNA-PS treatment (five out of five) and in three out of five animals after wash out.

In the kidney, no significant lesions were observed following 12 weeks of treatment with both tested compounds at 10 μmol/kg/week either 2 weeks after the last dose or after a 12-week wash-out period.

Overall, histopathological findings revealed only limited lesions in intensity and in extension, appearing more pronounced with the palm-tcDNA-PS compound.

### Evaluation of kidney toxicity

In order to study the potential renal toxicity that palm-tcDNA-ASO administration may induce due to their accumulation in proximal tubules, several biomarkers were examined including early kidney injury biomarkers (KIBs). Urines were collected from treated mice one week after the last dose or after a 12-week wash-out period, and levels of total protein and albumin were first measured and normalized with creatinine levels (Figure 8C). No changes in normalized total protein levels were found after any of the treatment. Urinary albumin levels appeared elevated after palm-tcDNA-PS treatment and went back to normal after the washout period. Interestingly, the palm-tcDNA-PO induced a slight decrease in albumin levels which were no longer different from WT levels. Moreover, treatment with palm-tcDNA-PO for 12 weeks did not affect the urinary levels of B2-microglobulin (B2M), renin, KIM-1, IP10, VEGF, cystatin C, EGF, neutrophil gelatinase-associated lipocalin (NGAL), clusterin and osteopontin (OPN) (Figure 8D). Palm-tcDNA-PS treatment on the other hand, induced a slight elevation of KIM-1 and renin levels (although not statistically significant for renin), which came back to normal levels after the 12-week washout-period.

## DISCUSSION

ASO therapeutics have gained increasing interest in the past few decades and several ASO drugs have reached market approval. However, the majority of systemically administered ASO are distributed to liver and kidney, limiting the therapeutic potential of this approach for neuromuscular diseases such as DM1 and DMD. Only the uncharged phosphorodiamidate morpholino (PMO) ASOs have so far been approved for the systemic treatment of DMD but their clinical benefit in DMD patients is still marginal. While PS-ASO can also reach muscle tissues, the doses required are generally very high and may result in toxicities associated with PS accumulation in liver and kidney. It is therefore therapeutically valuable to improve ASO activity in muscle tissues to better address clinical needs for NMDs. Many research efforts are currently focusing on extra hepatic tissue delivery and conjugation to fatty acid is an attractive way to improve delivery to muscle tissues. Previous studies have demonstrated efficient extrahepatic delivery of siRNAs (26) and gapmer ASOs (27,28) conjugated to fatty acid but this was never reported for splice-switching ASO (SSO) for which an additional barrier has to be overcome: the nuclear membrane. To be effective, SSOs have to be transported from the circulation to muscle cells nuclei and therefore pass the endothelium, the interstitial space, the plasma membrane and nuclear membrane. We have previously shown that tcDNA-ASO are promising ASO for NMD as they are capable of significantly reach muscle tissues and even cross the BBB at low levels after systemic delivery. We hypothesized that conjugation to palmitic acid would enhance their delivery to skeletal and cardiac muscles and improve their potency. We demonstrate here that palmitic acid improves the potency of both PO and PS-tcDNA ASO compared to their unconjugated counterparts. Palm-tcDNA-PS were 5-fold more potent than naked tcDNA-PS and, more strikingly, palm-tcDNA-PO were 50-fold more potent than tcDNA-PO. This improvement allows a significant reduction in the administered dose compared to our previously published studies where doses up to 40 μmol/kg of tcDNA-PS were injected. To further compare the potential of both conjugated compounds, we performed dose-response experiments in mice over a period of 3 months and showed that palm-tcDNA-PS induced significantly higher levels of exon skipping and dystrophin restoration than palm-tcDNA-PO at the dose of 10 μmol/kg. Interestingly at the lower dose, there were no statistical differences in potency between the two compounds. When calculating the ED50 for each compound, we found a 4-times lower ED50 for palm-tcDNA-PS than palm-tcDNA-PO. However, while the ED50 is a useful parameter to compare the potency of various compounds, it is mostly used in down-regulation studies to reflect the dose required to knock down 50% of the target mRNA. In the case of splice switching approaches and exon skipping in particular which aims at restoring the reading frame, the arbitrary 50% may not be very appropriate since much lower exon skipping levels may be therapeutically relevant for DMD patients. The level of dystrophin required for an effective clinical therapy in DMD patients is still under discussion. Several studies have suggested that approximately 20% of uniformly distributed dystrophin may be sufficient (29,30) and more recent work has shown that very low residual dystrophin quantity was associated with milder dystrophinopathy (31).

The range of dystrophin restoration obtained in this study was sufficient to improve functional outcomes in dystrophic mice and will likely be beneficial in patients. Moreover, despite the lower doses administered in this study (compared to our previous work (6,7)), tcDNA ASOs are still able to cross the BBB at low levels after conjugation to palmitic acid and partially restore expression of dystrophin protein in the brain, which improves emotional outcomes associated with the lack of brain dystrophin. Palm-tcDNA ASOs therefore represent particularly promising drugs for the systemic treatment of DMD, offering the possibility to address both the muscle pathology and the brain involvement associated with the absence of dystrophin in the brain.

While the palm-tcDNA-PS induces higher levels of exon skipping and dystrophin restoration than palm-tcDNA-PO and may appear as a more potent compound, several other considerations actually make the palm-tcDNA-PO a much more promising drug candidate. When calculating the ratio of efficacy over quantity detected in tissues, we found consistently better ratio for the palm-tcDNA PO suggesting better productive uptake for this compound (higher efficacy for less compound accumulated). This is particularly true after the washout period, where we found very little tcDNA-PO left in the tissues (about 10%) but still significant levels of exon skipping (about 63% of the levels detected before the 12wks wash-out). Considering the short half-life of DMD mRNA (32), this exon skipping level reflects the productive tcDNA-ASO fraction in the nucleus, which likely results from slow endosomal release over time. Despite showing higher accumulation in tissues, the ratio efficacy/quantity is lower for the palm-tcDNA-PS suggesting a lesser productive fraction for this compound which might be more retained in the interstitial space than its PO counterpart. Analysis of the metabolites performed by LCMS revealed that the palmitic acid conjugate was cleaved from the ASO found in tissues. Once freed from its palmitic acid conjugate, tcDNA-PO may be more advantageous than tcDNA-PS for cell trafficking and nuclear delivery.

More importantly, tcDNA-PO present a significant advantage in terms of toxicity compared to the tcDNA-PS. Our *in vitro* studies reveal that palm-tcDNA-PO has no impact on coagulation pathways nor complement activation in contrast with its PS counterpart. This was not observed *in vivo* because the doses administered were sufficiently low, but it is important to model potential toxicities that could occur in dose-ranging studies required for preclinical development. Moreover, considering that treatment for NMDs like DMD requires a lifetime medication, it is crucial to evaluate the impact of ASO accumulation in liver and kidney tissues to avoid potential long-term toxicities. No changes in hepatic or renal serum biomarkers were detected with any of the 2 compounds. However, some urinary biomarkers were slightly but statistically elevated after treatment with the palm-tcDNA-PS as opposed to palm-tcDNA-PO. Histopathological findings revealed only limited lesions, yet more pronounced with the palm-tcDNA-PS compound. Altogether our data suggest a better safety profile of the palm-tcDNA-PO than the palm-tcDNA-PS.

Moreover, interactions of PS-ASOs with proteins can influence their safety and it has been shown that binding of PS-ASOs to proteins can lead to nucleolar mislocalization of paraspeckle proteins and nucleolar stress, resulting in apoptotic cell death (33–35). While these subcellular aggregates may not all be detrimental, the use of PO-ASOs is an appealing strategy to overcome these PS-induced challenges.

The mechanism underlying the enhanced potency of palmitic acid conjugates is likely the improved binding to serum albumin as this was previously described for cEt (constrained ethyl) gapmers (19). Palm-cEt-PS gapmers were shown to be ∼5-fold more potent than their unconjugated PS counterparts, and this was associated with a 200-fold increase in affinity for albumin which is the most abundant plasma protein. An initial study comparing the conjugation of cEt gapmers to various lipids revealed that albumin affinity was correlated with the activity of fatty acid conjugates in skeletal and cardiac muscle and that palmitic acid showed the highest *in vivo* muscle activity (28).

Our findings with palm-tcDNA-PS corroborate the 5-fold improvement described in these studies and further demonstrate the potential of palmitic acid conjugation to PO-ASO with a 50-fold increase in potency compared to naked tcDNA-PO. Developing an effective PO-ASO represents a major milestone for the systemic treatment of DMD to avoid dose limiting toxicities in the clinic, which have often been a limitation in previous clinical development (as observed with drisapersen, a 2’OMePS and suvodirsen, a stereopure 2’Modified PS –ASO).

In conclusion, we report the detailed characterisation of palmitic acid conjugation to both PO and PS-tcDNA showing a significant increase in potency, associated with functional benefits and a particularly encouraging safety profile for the palm-tcDNA-PO compound. Further research improving specific and targeted delivery to muscle cells may provide added effectiveness, as exemplified by the success of GalNac conjugation for hepatocytes. Interestingly, while GalNac conjugated ASO showed 7- to 10-fold improved potency in rodents (36), they were found to be 30-fold more potent than the unconjugated ASO in humans (37). Extrapolating these results would suggest promising outcomes for lipid-conjugated tcDNA-ASO in patients.

## Supplementary Material

gkab1199_Supplemental_File
