# Supplementary material for: Palmitic acid conjugation enhances potency of tricyclo-DNA splice switching oligonucleotides
Source: Nucleic Acids Res. 2021 Dec 10;50(1):17–34. doi: 10.1093/nar/gkab1199 (PMC8754652; doi:10.1093/nar/gkab1199)
Supplement: gkab1199_Supplemental_File [file gkab1199_supplemental_file.pdf]

SUPPLEMENTARY DATA

Supplementary tables:

AONs	Ratio of tcDNA quantity										Average muscles
	TA	GAS	Quad	Tri	Bi	Dia	Heart	Spleen	Liver	Kidney	
tcDNA-PS / tcDNA-PO	13	25	19	19	21	4	8	8	36	27	16
Palm-tcDNA-PS / Palm-tcDNA-PO	4	3	3	2	3	10	3	9	2	1	4
Palm-tcDNA-PO / tcDNA-PO	22	38	32	56	31	4	16	12	66	156	28
Palm-tcDNA-PS / tcDNA-PS	6	4	4	5	5	12	6	14	4	7	6

Supplementary table 1. Calculation of the ratio of tcDNA amount quantified in the various tissues between the different compound, with the average indicated for muscles tissues. On average across the different muscle tissues, palm-tcDNA-PO accumulates 28 times more than tcDNA-PO.

AONs	Ratio of tcDNA efficacy							Average muscles
	TA	GAS	Quad	Tri	Bi	Dia	Heart	
tcDNA PS / tcDNA PO	61	7	6	3	2	10	57	21
Palm-tcDNA PS / Palm-tcDNA PO	3	2	8	5	1	1	1	3
Palm-tcDNA PO / tcDNA PO	123	14	5	3	7	13	187	50
Palm-tcDNA PS / tcDNA PS	7	5	7	5	3	2	4	5

Supplementary table 2. Calculation of the ratio of exon 23 skipping levels quantified in the various tissues between the different compound, with the average indicated for muscles tissues. On average across the different muscle tissues, palm-tcDNA-PO induces 50 times higher exon skipping levels than tcDNA-PO.

		TA	GAS	QUAD	TRI	Bi	DIA	Heart	
Palm-tcDNA-PO	Skip efficiency (%)	2,6	6,9	3,0	1,1	1,8	1,2	3,9	**Muscles mean**
**2µmol/kg**	Quantity in tissues (µg/g)	0,5	1,3	0,6	0,6	2,1	0,4	3,2	
**12 wks**	**RATIO**	**5,3**	**5,2**	**4,7**	**1,7**	**0,9**	**3,2**	**1,2**	**3,2**
		**TA**	**GAS**	**QUAD**	**TRI**	**Bi**	**DIA**	**Heart**	
Palm-tcDNA-PO	Skip efficiency (%)	6,3	8,2	4,4	2,4	7,2	2,7	7,7	**Muscles mean**
**4µmol/kg**	Quantity in tissues (µg/g)	1,0	4,7	1,7	1,0	11,5	2,6	8,2	
**12 wks**	**RATIO**	**6,2**	**1,7**	**2,6**	**2,4**	**0,6**	**1,1**	**0,9**	**2,2**
		**TA**	**GAS**	**QUAD**	**TRI**	**Bi**	**DIA**	**Heart**	
Palm-tcDNA-PO	Skip efficiency (%)	12,6	13,6	15,3	8,3	20,7	8,0	13,8	**Muscles mean**
**10µmol/kg**	Quantity in tissues (µg/g)	3,0	7,2	5,5	4,3	10,9	4,3	20,8	
**12 wks**	**RATIO**	**4,2**	**1,9**	**2,8**	**1,9**	**1,9**	**1,9**	**0,7**	**2,2**
								Mean palm-tcDNA-PO	***2,5***
		**TA**	**GAS**	**QUAD**	**TRI**	**Bi**	**DIA**	**Heart**	
Palm-tcDNA-PS	Skip efficiency (%)	2,1	8,3	2,8	2,7	2,3	1,7	1,5	**Muscles mean**
**2µmol/kg**	Quantity in tissues (µg/g)	1,0	1,6	0,9	1,3	4,0	1,2	5,4	
**12 wks**	**RATIO**	**2,1**	**5,2**	**3,0**	**2,2**	**0,6**	**1,5**	**0,3**	**2,1**
		**TA**	**GAS**	**QUAD**	**TRI**	**Bi**	**DIA**	**Heart**	
Palm-tcDNA-PS	Skip efficiency (%)	4,7	21,2	9,9	6,7	4,7	6,8	5,0	**Muscles mean**
**4µmol/kg**	Quantity in tissues (µg/g)	1,9	8,3	2,8	2,0	25,6	14,3	25,7	
**12 wks**	**RATIO**	**2,4**	**2,6**	**3,5**	**3,4**	**0,2**	**0,5**	**0,2**	**1,8**
		**TA**	**GAS**	**QUAD**	**TRI**	**Bi**	**DIA**	**Heart**	
Palm-tcDNA-PS	Skip efficiency (%)	32,0	56,4	37,1	33,7	41,9	28,6	19,6	**Average accross**
**10µmol/kg**	Quantity in tissues (µg/g)	23,9	28,0	18,1	25,7	38,8	54,4	81,5	**tissue**
**12 wks**	**RATIO**	**1,3**	**2,0**	**2,1**	**1,3**	**1,1**	**0,5**	**0,2**	**1,2**
								Mean palm-tcDNA-PS	***1,7***

Supplementary table 3. Calculation of the ratio of the exon skipping levels on ASO quantity per tissue, representing the therapeutic potential of each compound (i.e. the compound inducing the highest levels of exon skipping with minimal tcDNA accumulation).

Supplementary Figures:

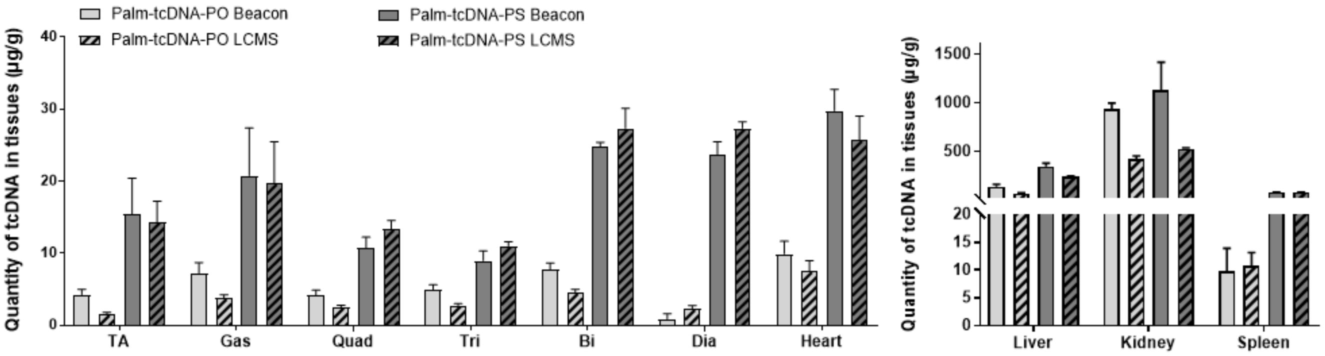

Figure S1. Quantification of palm-tcDNA-ASO content by fluorescent hybridization assay and LCMS reveals similar results. Quantification of palm-tcDNA-ASO content in various *mdx* mice tissues 2 wks after the end of the 4-wk dosing regimen (palm-tcDNA-PO or palm-tcDNA-PS at 10 µmol/kg/week) by fluorescent hybridization assay (Beacon) and by LCMS. (TA: tibialis anterior, Gas: gastrocnemius, Quad: quadriceps, Tri: triceps, Bi: biceps; Dia: diaphragm). Results are expressed as mean ± SEM; n=4 for palm-tcDNA-PO treated group and n=3 for palm-tcDNA-PS treated group.

A

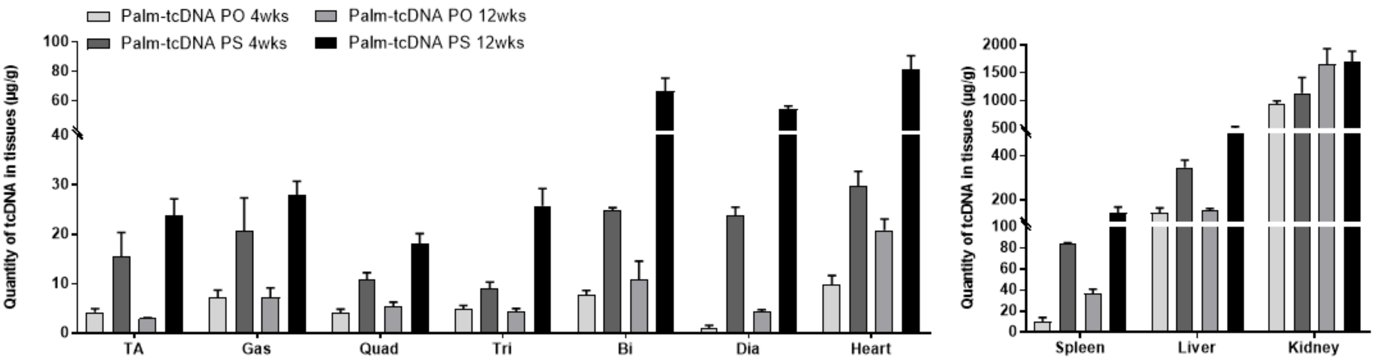

ASOs	Ratio of tcDNA quantity 12wks/4wks										Average muscles
	TA	GAS	Quad	Tri	Bi	Dia	Heart	Spleen	Liver	Kidney	
Palm-tcDNA-PO	0,7	1,0	1,3	0,9	1,4	1,9	2,1	3,7	1,1	1,8	1,3
Palm-tcDNA-PS	1,6	1,3	1,7	2,9	1,6	2,3	2,7	2,7	1,5	1,5	2,0

B

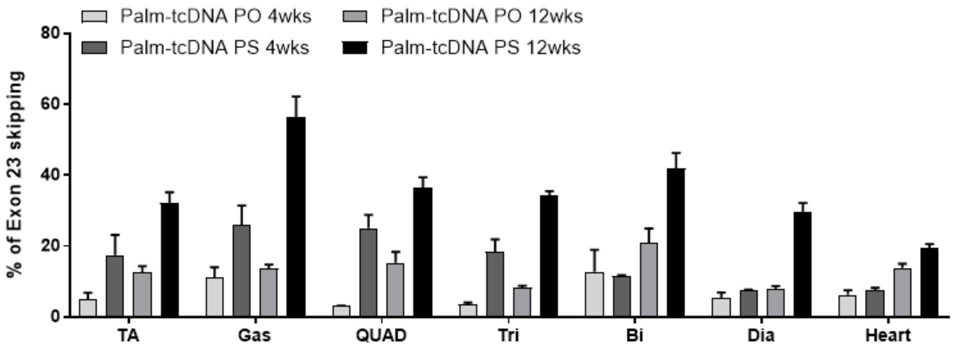

ASOs	Ratio of tcDNA efficacy 12wks/4wks							Average muscles
	TA	GAS	Quad	Tri	Bi	Dia	Heart	
Palm-tcDNA-PO	2	1	5	2	2	1	2	2,4
Palm-tcDNA-PS	2	2	1	2	4	4	3	2,5

C

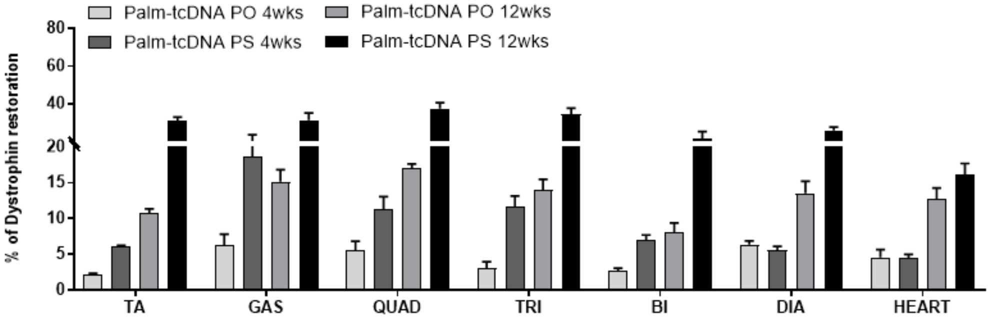

ASOs	Ratio of % dystrophin restoration 12wks/4wks							Average muscles
	TA	GAS	Quad	Tri	Bi	Dia	Heart	
Palm-tcDNA-PO	5	2	3	5	3	2	3	3,3
Palm-tcDNA-PS	5	2	3	3	3	5	4	3,5

Figure S2. Long term treatment enhances biodistribution, efficacy and dystrophin rescue of palm-tcDNA-ASO. (A) Quantification of palm-tcDNA-ASO content in various *mdx* mice tissues 2 wks after the end of a 4-wk or a 12-wk dosing regimen (palm-tcDNA-PO or palm-tcDNA-PS at 10 µmol/kg/week) by fluorescent hybridization assay. Ratio are calculated between long (12-wk) and short (4-wk) term treatment for each palm-tcDNA-ASO. **(B)** Quantification of exon 23 skipping levels by taqman RT-qPCR in the different muscle tissues after short (4-wk) or long (12-wk) term treatment and ratio calculation. **(C)** Quantification of dystrophin restoration by western blot in the different muscle tissues after short (4-wk) or long (12-wk) term treatment and ratio calculation. (TA: tibialis anterior, Gas: gastrocnemius, Quad: quadriceps, Tri: triceps, Bi: biceps; Dia: diaphragm). Results are expressed as mean ± SEM; n=4 mice per group.

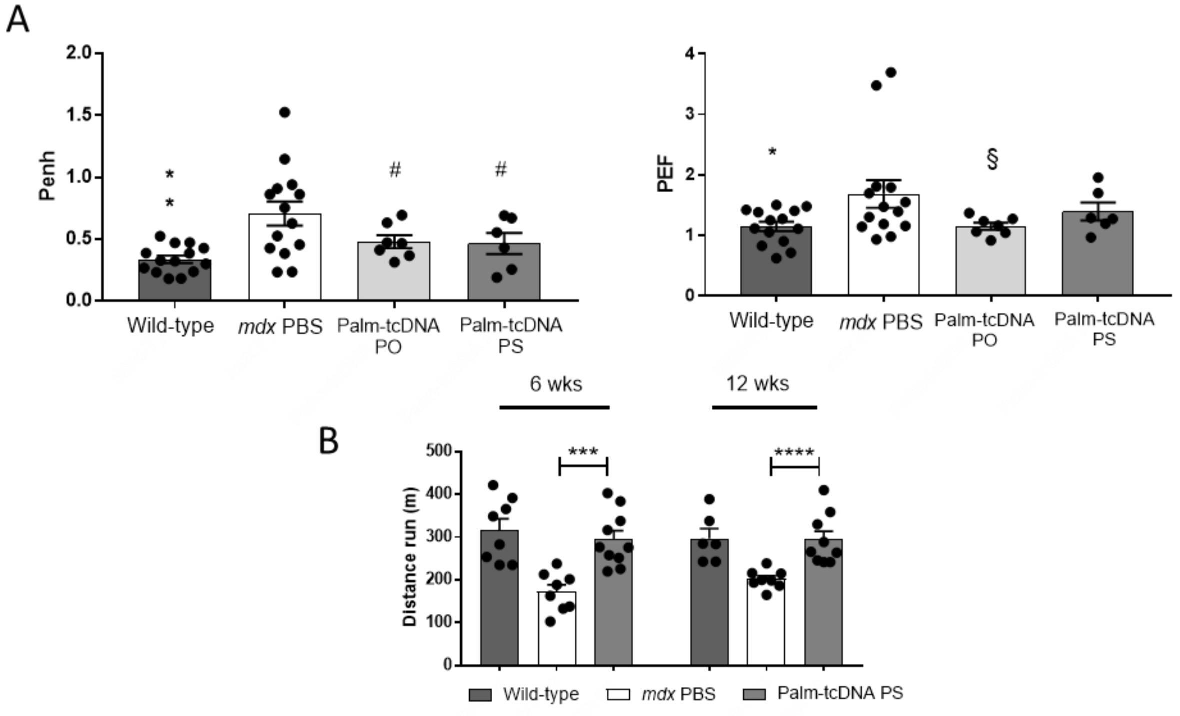

Figure S3. Long term treatment of palm-tcDNA-ASO improves dystrophic pathology in *mdx* mice. (A) Respiratory function of *mdx* mice treated with 10µmol/kg/wk of palm-tcDNA for 12 weeks (n = 6 per group) compared to *mdx* control (n = 14) and *wild-type* mice (n = 14). Penh and PEF are shown. Error bars are means ±sem. *P < 0.05, **P < 0.01, §P = 0.06 compared to *mdx* control mice (Mann–Whitney U tests). # ns compared to *wild-type* mice.

(B) Running distance during incremental speed running test until exhaustion after 6 weeks and 12 weeks of treatment with Palm-tcDNA-PS compared to *mdx* PBS treated control mice. Results are expressed as mean ± SEM; n= 8 mice per group. ***P < 0.001, ****P < 0.0001 compared to *mdx* control mice (Mann–Whitney U tests).

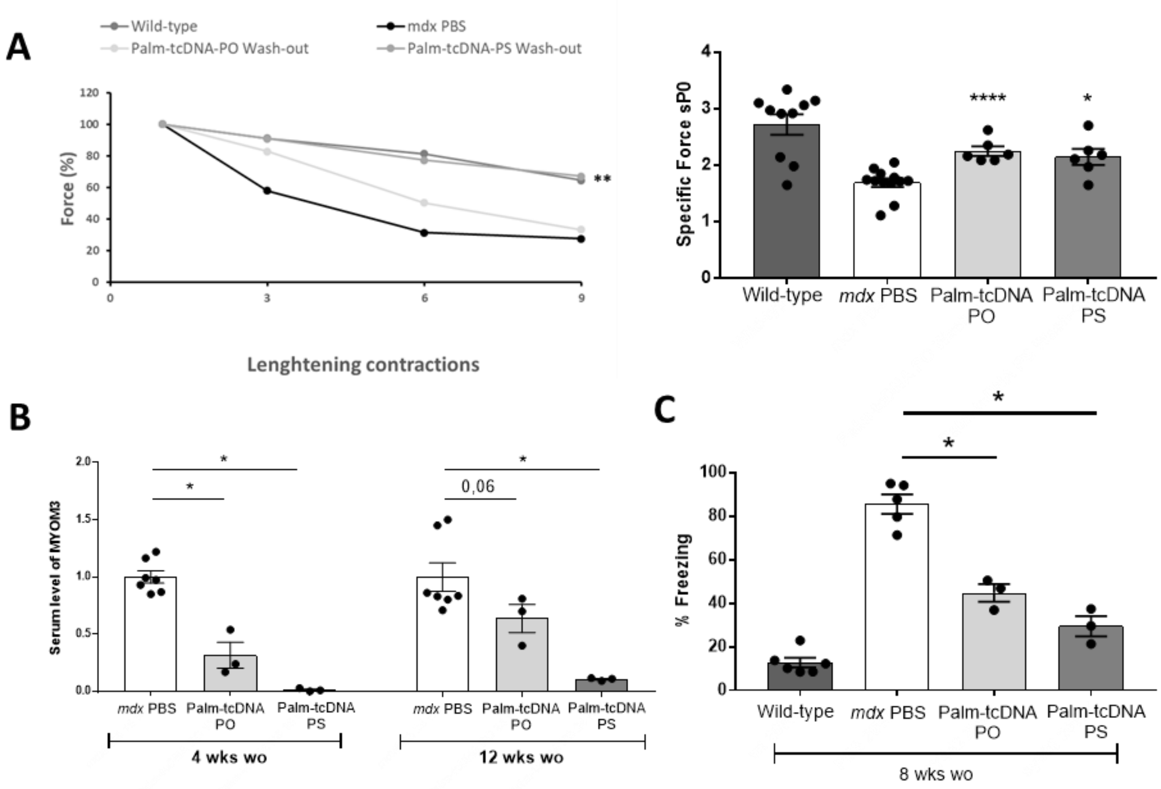

Figure S4. Palm-tcDNA-ASO effects on muscle function are maintained after several weeks of wash-out period. (A) Percentage of force drop following a series of eccentric contractions (left panel) and maximal specific force (sP0) (right panel) measured on semi-isolated tibialis anterior muscles from *mdx* mice treated with palm-tcDNA-PO or palm-tcDNA-PS (n=6 mice per group) 12 weeks after the end of the treatment (12 weeks at 10µmol/kg/wk) and compared to WT (n=10 mice) and PBS control mdx mice (n=12 mice). Results are expressed as mean ± SEM. *p < 0,05 ; **p < 0,01; ****p < 0.0001 compared to PBS treated controls (Mann-Whitney U tests). **(B)** Quantification of MYOM3 levels by western blot in serum collected 4 and 12 weeks after the end of the 12-wk dosing regimen (indicated as 4 wks wo and 12 wks wo respectively on the graph). *Mdx* mice treated with palm-tcDNA-PO or palm-tcDNA-PS at 10µmol/kg/week (n=3 mice/group) were compared to PBS control *mdx* mice (n=7 mice/group). Results are expressed as mean ± SEM. *p < 0.05 (Mann-Whitney U tests). **(C)** Restraint-induced unconditioned fear responses measured

8 weeks after the end of the 12-wk dosing regimen at 10µmol/kg/week and expressed as a percentage of freezing time in wild-type (n=6 mice) and *mdx* mice treated with PBS (n=5 mice), palm-tcDNA-PO or palm-tcDNA-PS (n=3 mice/group). Results are expressed as mean ± SEM; *p < 0.05 (Mann-Whitney U tests)

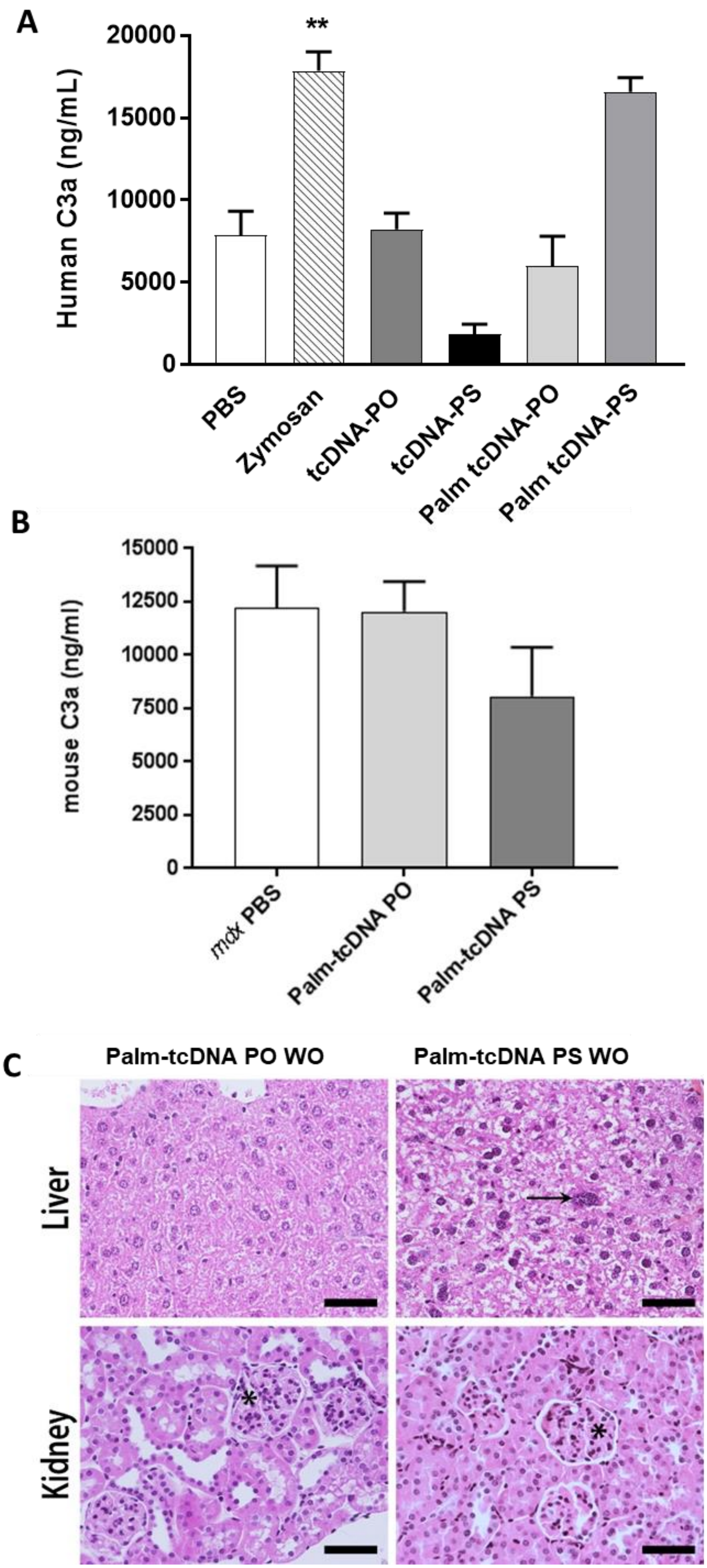
A
**
Human C3a (ng/mL)
20000
15000
10000
5000
0
PBS
Zymosan
tcDNA-PO
tcDNA-PS
Palm tcDNA-PO
Palm tcDNA-PS
B
mouse C3a (ng/ml)
15000
12500
10000
7500
5000
2500
0
mdx PBS
Palm-tcDNA PO
Palm-tcDNA PS
C
Palm-tcDNA PO WO
Palm-tcDNA PS WO
Liver
Kidney
*
*

Figure S5. Complement activation. (A) Human C3a anaphylotoxin was analysed by elisa in human serum samples incubated with 0,2 mg/ml of tcDNA-PO (n=3), tcDNA-PS (n=8), palm-tcDNA-PO (n=6) or palm-tcDNA-PS (n=3). PBS (n=14) and Zymosan (n=12) were used as negative and positive control respectively. Results are expressed as mean ± SEM. ** $p<0.01$compared to PBS (One-way annova). **(B)** Mouse C3a anaphylotoxin was analysed by elisa in mouse serum samples collected 1h after administration of 10µmol/kg of palm-tcDNA-PO, palm-tcDNA-PS or PBS. Results are expressed as mean ± SEM, n=5 mice per group. **(C)** Hemalun-Eosin-Saffron staining on liver (upper panel) and kidney (lower panel) sections of *mdx* mice 12 weeks after the end of the 12-wk dosing regimen with palm-tcDNA-PO or palm-tcDNA-PS at 10µmol/kg/week. An increase in size heterogeneity of hepatocyte nuclei was present with notably numerous binucleated cells and meganucleation (arrow). Scale bar = 50 µm.
